# Discriminating between competing models for the allosteric regulation of oncogenic phosphatase SHP2 by characterizing its active state

**DOI:** 10.1016/j.csbj.2021.10.041

**Published:** 2021-11-03

**Authors:** Paolo Calligari, Valerio Santucci, Lorenzo Stella, Gianfranco Bocchinfuso

**Affiliations:** Dipartimento di Scienze e Tecnologie Chimiche, Università di Roma Tor Vergata, Rome, Italy

**Keywords:** SHP2 regulatory mechanism, Replica exchange molecular dynamics simulations, Inter-domain dynamics, Protein flexibility, BTLA, B and T lymphocyte attenuator, CTLA-4, cytotoxic T lymphocyte-associated antigen 4, FRET, Förster resonance energy transfer, JMML, juvenile myelomonocytic leukemia, MD, molecular dynamics, NS, Noonan syndrome, NSML, Noonan syndrome with multiple lentigines, PD-1, programmed cell death protein 1, PDB, protein data bank, PMF, potential of mean force, PTP, protein tyrosine phosphatase, pY, phosphorylated tyrosine, REMD, replica exchange molecular dynamics, RMSF, root mean square fluctuation, RMSD, root mean square deviation, RTK, receptor tyrosine kinase, SASA, solvent accessible surface area, SAXS, small angle X-ray scattering, SH2, Src homology 2, SHP2, Src homology 2 domain-containing phosphatase 2, SIRPalpha, signal regulatory protein alpha

## Abstract

The Src-homology 2 domain containing phosphatase 2 (SHP2) plays a critical role in crucial signaling pathways and is involved in oncogenesis and in developmental disorders. Its structure includes two SH2 domains (N-SH2 and C-SH2), and a protein tyrosine phosphatase (PTP) domain. Under basal conditions, SHP2 is auto-inhibited, with the N-SH2 domain blocking the PTP active site. Activation involves a rearrangement of the domains that makes the catalytic site accessible, coupled to the association between the SH2 domains and cognate proteins containing phosphotyrosines. Several aspects of this transition are debated and competing mechanistic models have been proposed. A crystallographic structure of SHP2 in an active state has been reported (PDB code 6crf), but several lines of evidence suggests that it is not fully representative of the conformations populated in solution. To clarify the structural rearrangements involved in SHP2 activation, enhanced sampling simulations of the autoinhibited and active states have been performed, for wild type SHP2 and its pathogenic E76K variant. Our results demonstrate that the crystallographic conformation of the active state is unstable in solution, and multiple interdomain arrangements are populated, thus allowing association to bisphosphorylated sequences. Contrary to a recent proposal, activation is coupled to the conformational changes of the N-SH2 binding site, which is significantly more accessible in the active sate, rather than to the structure of the central β-sheet of the domain. In this coupling, a previously undescribed role for the N-SH2 BG loop emerged.

## Graphical abstract

**Figure:**
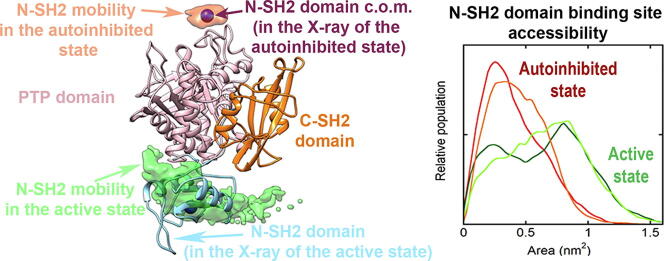

## 1 Introduction

The Src homology 2 domain-containing phosphatase 2 (SHP2) was the first protein tyrosine phosphatase for which an oncogenic role was demonstrated [1]. This protein mediates signal transduction downstream of various receptor tyrosine kinases (RTKs), being required for full activation of the RAS-MAP kinase pathway [2] and modulating signal transduction also through other cascades, such as PI3K-AKT and JAK-STAT [3]. Somatic gain of function mutations of *PTPN11*, the gene coding for SHP2, cause 35% of juvenile myelomonocytic leukemia (JMML) cases and are found in other hematologic malignancies and tumors [1], [3], [4], [5]. In addition, germline missense mutations in *PTPN11* occur in ∼50% of individuals with Noonan syndrome, a JMML-prone developmental disorder [6]. SHP2 activity is required for survival of receptor tyrosine kinases (RTK)-driven cancer cells [7] and is involved in intrinsic and acquired resistance to targeted cancer drugs [8], [9], [10], [11], [12], [13], [14], [15]. Finally, SHP2 mediates activation of immune checkpoint pathways, such as programmed cell death 1 (PD-1), B and T lymphocyte attenuator (BTLA), cytotoxic T lymphocyte-associated antigen 4 (CTLA-4) and signal regulatory protein alpha (SIRPalpha) [16], [17], [18] and it is involved in the induction of gastric carcinoma by *H. pylori* [19], [20]. For all these reasons, SHP2 is now a central target in cancer therapy [21].

**Fig. 1:**
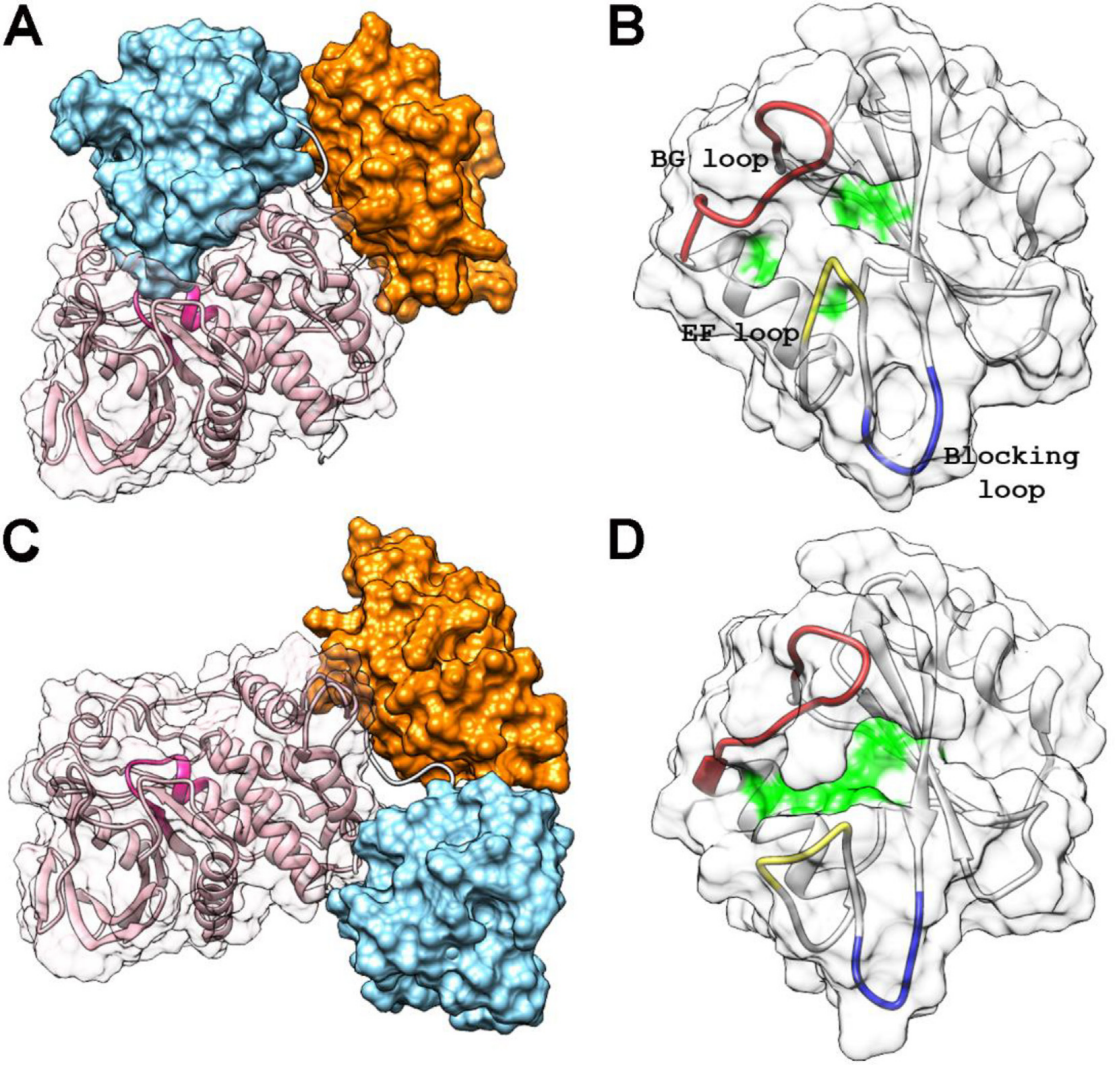
Structure of SHP2 in the autoinhibited and active state. Panels A and B refer to the autoinhibited state of the WT protein (PDB code 4dgp), while panels C and D report the structure of the E76K mutant in the active state (PDB code 6crf). Panels A and C report the whole protein, with the N-SH2, C-SH2 surfaces colored in light blue and orange respectively, and the PTP catalytic domain reported as a pink ribbon. The catalytic site on the PTP domain (residues 457–467) is colored in dark pink. The PTP is also reported as a semitransparent white surface. The structures of the N-SH2 domain are reported in more detail in panels B and D. Under the semitransparent white surface, the backbone is reported with the BG (residues 84–96), EF (66–69) and blocking, or DE, (58–62) loops colored in red, yellow and blue, respectively. On the semitransparent surface, residues 54, 65 and 81 are evidenced in green. These amino acids are involved in the interaction with the phosphopeptide residues located at position +1, +3 and +5 with respect to the pY, which insert into the binding groove [22]. (For interpretation of the references to color in this figure legend, the reader is referred to the web version of this article.)

SHP2 comprises two Src homology 2 (SH2) domains, called N-SH2 and C-SH2, followed by the catalytic protein tyrosine phosphatase (PTP) domain, and a C-terminal tail. SH2 domains are recognition elements that bind protein sequences containing a phosphorylated tyrosine (pY) [22]. In SHP2, they also have an essential role in modulating the catalytic activity of the protein. In the crystallographic structure of SHP2 in the inactive state (e.g. PDB code 4dgp), one of the loops of the N-SH2 domain (DE, or “blocking” loop) blocks the catalytic site of the PTP domain, so that no activity is possible (Fig. 1A). At the same time, the phosphopeptide binding site of the N-SH2 domain is closed, due to the conformation of loops EF and BG, so that signaling partners cannot bind in this conformation (Fig. 1B). By contrast, structures of the isolated N-SH2 domain (e.g. PDB code 1ayd) show that lack of interaction with the PTP domain causes opening of the phosphopeptide binding groove and a conformational change in the DE loop, which makes its insertion in the PTP active site impossible.

**Fig. 2:**
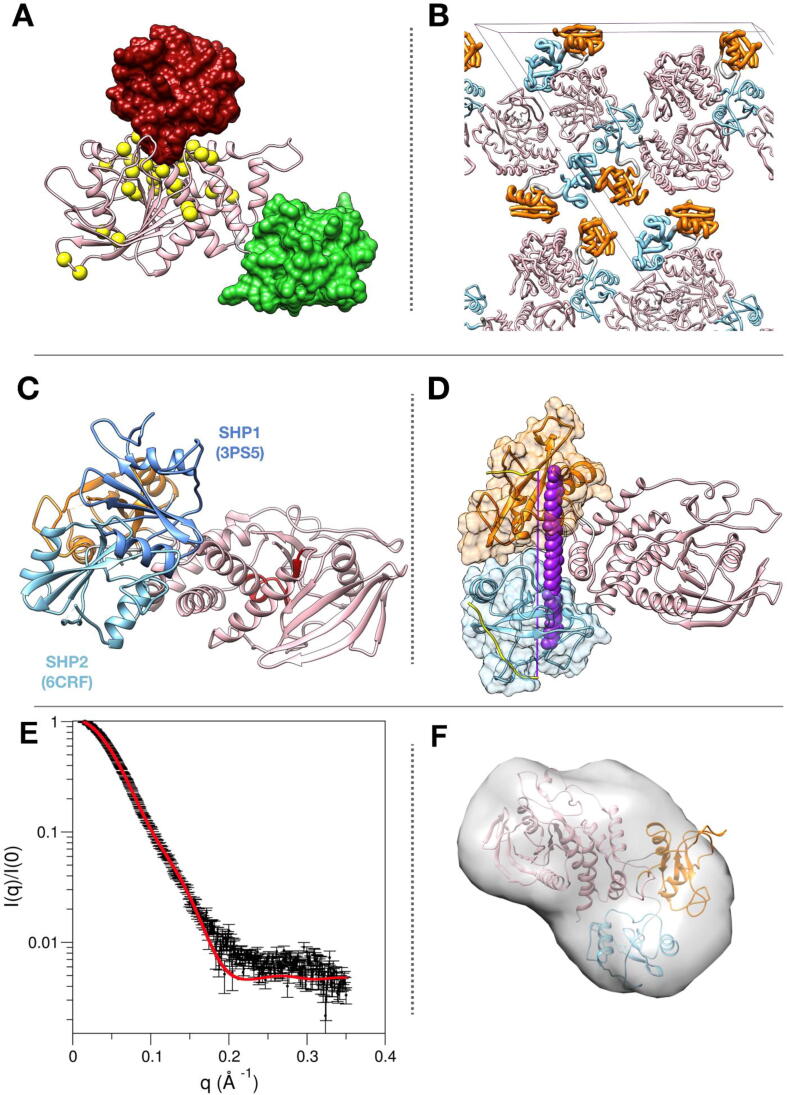
Analysis of the crystallographic structure of SHP2 (E76K) in the active state. *Panel A)* localization in the PTP domain of residues affected by pathogenic mutations linked to cancer, Noonan syndrome (NS) or Noonan syndrome with multiple lentigines (NSML). The C_α_ atoms of affected residues are shown as yellow spheres (residues 285, 289, 308, 402, 491, 502, 503, 507, 510 for cancer, 221, 256, 258, 261, 262, 265, 268, 282, 285, 308, 309, 347, 395, 396, 409, 411, 428, 491, 501, 502, 503, 504, 507 for NS and 279, 461, 464, 468, 506, 510 for NSML). The N-SH2 domain in the autoinhibited and active structures of the whole protein (PDB codes 4dgp and 6crf, respectively) are reported as red and green surfaces, respectively. *Panel B)* unit cell of the crystallographic structure of E76K in the active conformation (PDB 6crf). N-SH2 (light blue) and C-SH2 (orange) domains are shown in worm-like representation, with a backbone radius proportional to crystallographic Debye-Waller factors. The two N-SH2 domains belonging to the same asymmetric unit exhibit a significantly different mobility. *Panel C)* Crystallographic structure of SHP2 in the active state (PDB code 6crf) [37]. For comparison, the N-SH2 position with respect to PTP in the crystallographic structure of SHP1 in the active state (PDB code 3ps5) [68] is reported, too. N-SH2 domains are reported in different hues of blue. C-SH2 and PTP domains from 6crf are colored in orange and pink, respectively. The PTP active site is reported in red. *Panel D)* A high affinity biphosphorylated peptide, with optimized length [28] is not able to bind the two SH2 domain in the crystallographic structure of the active state. The two phosphopeptides were docked in the SH2 domains of the crystallographic structure of E76K in the active state. The (Ahx)_4_ linker included in the bisphosphorylated peptide used in the experiments is shown in purple. Even in a fully extended conformation going directly from one binding region to the other (and thus passing hypothetically through the protein structure), it is clearly not long enough to join the two phosphopeptides. *Panel E)* SAXS profile calculated from the crystallographic structure of the E76K variant in the active conformation (PDB code 6crf), fitted to experimental data from [45] using FOXS [69], *Panel F*) Molecular envelope obtained from the SAXS profile of the E76K variant, compared to the crystal structure for E76K in the active conformation (PDB code 6crf), using the ATSAS package (DAMMIN and SUPCOMP programs) [70]. (For interpretation of the references to color in this figure legend, the reader is referred to the web version of this article.)

Based on these structural data, a model of SHP2 regulation was proposed, where activation and association of the SH2 domains to binding partners are allosterically coupled: SHP2 binding to cognate proteins favors an active state, where the N-SH2 domain is displaced from the catalytic site and the protein is active [23]. Indeed, biochemical data show that association to phosphorylated sequences through the SH2 domains causes a dramatic activation of the phosphatase [24], [25], [26], [27], [28]. This picture was supported also by simulations [29], [30], [31] and by the thorough characterization of pathogenic *PTPN11* mutations [32], [33], [34], [35], [36]. Most mutations cluster at the N-SH2/PTP interface (Fig. 2A), destabilizing the inter-domain interactions and thus causing constitutive activation of the phosphatase [29], [32], [34]. More recently, a crystallographic structure was reported for the JMML-causing SHP2 E76K variant (PDB code 6crf) [37], which is fully activated in the basal state and therefore populates predominantly the active state (Fig. 1C, D). The X-ray structure is consistent with the hypothesis discussed above: the N-SH2 domain is far removed from the active site, and its phosphopeptide binding groove is open.

While the overall framework of the allosteric process controlling SHP2 activity is established, several essential aspects are still debated. Different mechanisms have been proposed for the relationship between protein–protein interactions and catalytic activation. One model [23] foresees that association to binding partners causes a conformational transition in the N-SH2 domain, which induces its detachment from the catalytic site and activation of the phosphatase (induced fit mechanism). This hypothesis contrasts with the fact that the grove in the N-SH2 domain, which binds the peptide sequence C-terminal to the pY residue, is inaccessible in the structure of the autoinhibited state. Recently, two hypotheses tried to reconcile this contradiction. Carlomagno and coworkers [38] suggested that the first step in the interaction could be the association of the pY residue to its binding pocket (always accessible), which in turn would cause the opening of the EF and BG loops and favor complete binding of the phosphopeptide. On the other hand, Anselmi and Hub considered this multistep mechanism unnecessary, since the motions of the N-SH2 domain would cause transient opening of the EF and BG loops and of the posphopeptide binding site, even in the autoinhibited state of the phosphatase [39]. Alternatively to the induced fit model, based on the observation that the basal activity of SHP2 is low but significant, we [29] and others [40] hypothesized that even in the basal state the protein populates transiently the open, active conformation, where the N-SH2 domain is dissociated from the active site (which is therefore accessible to substrates), and that this state is stabilized by association of the SH2 domains to their binding partners, in a conformational selection mechanism. In agreement with this hypothesis, *PTPN11* mutations destabilizing the autoinhibited state cause an increased affinity of SHP2 for its binding partners, in addition to catalytic activation [29], [32], [34], [41].

An additional element of debate regards the conformational switch taking place in the N-SH2 domain. The original hypothesis, based on the crystallographic structures [23], considered a coupling between the DE loop (inserting in the catalytic site of the PTP domain in the autoinhibited conformation) and the EF loop, controlling access to the binding site. According to this model, binding of a phosphopeptide to the domain would stabilize an open conformation for the EF loop, which, in turn, would induce a conformation of the DE loop incompatible with insertion in the PTP active site. At the same time, dissociation of the DE loop from the PTP domain would induce a conformational transition in the EF loop, causing opening of the active site and increased binding affinity. This model explains both the activation process and the increased affinity for binding partners of basally activated pathogenic mutants and it is structurally very straightforward, since the two loops are directly connected by the E β-strand. Finally, this model was supported by early MD simulations of the isolated N-SH2 domain and of the whole protein in the autoinhibited state [30], [31], [42], which identified the conformation of Y66 as the main switch controlling the structural transition. An extended backbone conformation for Y66 would be associated to a closed N-SH2 phosphopeptide binding site, while a left-handed helical backbone conformation would correspond to an open cleft [30]. Very recently, this model was challenged by Anselmi and Hub [39], [43] based on MD simulations of the isolated N-SH2 domain and autoinhibited structure. In their model, the dynamics of the EF and BG N-SH2 loops is not correlated to the activation equilibrium of the protein, which would rather be controlled by the conformation of two β-strands in the core of the N-SH2 domain, coupled to binding of specific phosphopeptide sequences.

Finally, a third aspect that needs to be clarified is the relevance of the crystallographic structure of the active state (PDB code 6crf) for the actual behavior of the enzyme in solution. In principle, the crystallographic structure of the E76K mutant could be representative for the active state populated to different degrees by WT SHP2 and by its pathogenic mutants, even in the absence of bound phosphopeptides. In the X-ray conformation, the overall arrangement of the domains is compact, with the N-SH2 domain interacting with the PTP side opposite to the catalytic cleft. Based on several lines of experimental evidence, we hypothesize that this structure is not representative of the conformations sampled by active SHP2 in solution:

i) the compact conformation reported for the active state in the E76K SHP2 crystal, and in particular the position of the N-SH2 domain, is stabilized by crystallographic contacts [37] (Fig. 2B). In addition, according to crystallographic Debye-Waller factors, the two N-SH2 domains belonging to the same asymmetric unit exhibit varying degrees of mobility, suggesting a different role of the constraints imposed by crystal packing (Fig. 2B and Fig. S1);
ii) a crystallographic structure of the active state has been reported also for the closely related protein SHP1 (PDB code 3ps5). While this structure, too, is compact, the position of the N-SH2 domain differs from that observed in the active structure of SHP2 (Fig. 2C). This discrepancy might be related to the fact that crystal packing is different for the two structures;
iii) in the case of a compact structure of the active state, the interface formed by the N-SH2 and PTP domains would provide an additional site to modulate the allosteric equilibrium. In principle, a shift in the active/inactive state equilibrium, inducing an increase in SHP2 activity, could be caused by mutations stabilizing the active state, in addition to amino acidic substitutions destabilizing the autoinhibited conformation (as commonly observed). However, no PTP residue affected by pathogenic mutations is located at the N-SH2/PTP interface indicated by the crystallographic structure of the active state (Fig. 2A). Hundreds of different pathogenic *PTPN11* mutations have been identified (in thousands of patients) [21], [32], and the lack of PTP mutations at the interface of the active structure is unexpected;
iv) small angle X-ray scattering (SAXS) measurements in solution conducted by different groups have been reported for SHP2 in the autoinhibited state [20], [37], [44], [45], [46], and in a partially or completely active state, induced by activating mutations [37], [44], [45], [46], or by association to phosphorylated sequences [20]. While data on the unstimulated WT protein, which is predominantly inactive, are essentially consistent with the closed crystallographic structure, studies on constitutively active variants or on activated WT SHP2 are undiscriminating and contradictory. LaRochelle et al. [37], who determined the X-ray structure for the E76K variant (PDB code 6crf) also reported SAXS data that were consistent with the crystallographic conformation. However, another set of SAXS data for the same variant, with a more extended range of scattering vector values, was reported by the Kern group [45] and was fitted with a model in which the N-SH2 and PTP domains are not in contact. A similar interpretation was provided for SAXS data on SHP2 bound to an activating monophosphorylated peptide (through the N-SH2 domain) [20]. Indeed, we found that the Kern data are not fully compatible with the 6crf structure, as shown both by the poor fit of the scattering data ($\chi^{2}$ = 3.7) and by the envelope calculations (normalized spatial discrepancy, NSD = 1.03, with 75 atoms not fitting in the envelope) (Fig. 2E and F);
v) additional clues on the active structure of SHP2 in solution emerged from other experimental techniques. NMR chemical shifts were indicative of an active state where the N-SH2 domain is totally dissociated from the PTP domain, while the C-SH2 domain is rotated (with respect to the autoinhibited conformation), but still interacts with the catalytic domain [45]. Two recent studies, employing Förster resonance energy transfer (FRET) were indicative of a continuum distribution of interprobe distances between the autoinhibited and active states [46], [47] with the population of intermediate states [46];
vi) the two SH2 domains associate to bisphosphorylated sequences of signaling partners. The distance between the two phosphorylated sequences that leads to an optimized binding affinity has been determined [28], and it corresponds to four 6-aminohexanoic acid residues, from the +5 residue of the N-terminal phosphopeptide to the −2 residue of the C-terminal peptide (residues are number counting from the pY), which in extended conformation span 41 Å. It is worth mentioning that even much shorter bisophosphorylated peptides are able to activate SHP2, even if with lower efficiency [28]. The arrangement of the two SH2 domains in the crystallographic structure of the E76K variant in the active state would not allow such a peptide to bind both domains, even in a completely stretched conformation (Fig. 2D). This is in contrast with the observation that binding to bisphosphorylated peptides stabilizes the active state of SHP2;
vii) Clemens et al. [48] recently determined the distance that the closely related protein SHP1 can reach from where it binds (SH2 domains) to where it exerts its catalytic activity (PTP domain), and found a value of 13 nm, to be compared with a distance of 5 nm in the crystal structure of the active state and of 20 nm if fully extended interdomain linkers are considered [48].

Taken together, these observations, strongly suggest that the SHP2 active structure obtained in the crystal state may not be fully representative of the conformational landscape populated in solution. The real nature of the open, active state of SHP2 (either caused by activating mutations or by association to binding partners through the SH2 domains) remains to be determined.

As discussed above, SHP2 is a central therapeutic target. Unfortunately, drugs inhibiting its catalytic site are affected by poor selectivity and low bioavailability [21], [49], [50]. Allosteric inhibitors, stabilizing the closed conformation by binding at the interdomain interface, have been much more successful and several molecules of this class are now in clinical trials [21], [50]. However, they are poorly active on pathogenic mutants, since the allosteric pocket is lost in the active conformation [37], [47]. We recently proposed an additional strategy, aimed at inhibiting SHP2 protein–protein interactions [41]; however, a full characterization of the conformational landscape populated by SHP2 could provide novel mechanisms for allosterically modulating the activity of this phosphatase [51], [52].

Molecular dynamics (MD) simulations are particularly suited to characterize the conformational heterogeneity of proteins in solution, and were employed in several articles to characterize various aspects related with SHP2 regulation (an overview of these studies is provided in Table S1). However, standard or enhanced sampling MD simulations of SHP2 pathogenic variants or of SHP2/phosphopeptide complexes starting from the autoinhibited state observed no dramatic variations of the interdomain arrangement [29], [39], [53], [54], [55], [56], [57], [58]. This finding is not surprising, considering that stopped-flow measurements [45] and single molecule FRET experiments [46], [47] indicate that interconversion between the autoinhibited and active states takes place in the seconds time-range; transitions with such high barriers are not amenable to sampling by currently reachable MD timescales.

In the present paper, we report MD simulations starting from the crystallographic structure of the E76K variant in the active state, using enhanced sampling replica exchange MD (REMD) simulations. The simulations have been carried out on the WT protein and on the E76K variant, in the absence of activating phosphopeptides. Under these conditions, experimental data show that the WT protein mainly populates the autoinhibited state, while E76K is fully active [29], [32]. For comparison, simulations have been performed also starting from the inactive state. While we did not expect to sample the transition between the two states even at the highest temperature of our REMD simulations (400 K), for the reasons discussed above, our goal has been to elucidate the dynamical features of SHP2 in its autoinhibited and active states, and to characterize how the different states affect the conformation of the N-SH2 domain and its propensity to associate to its binding partners. Our results indicate that the crystallographic interdomain arrangement of the active state is unstable in solution and that the N-SH2 domain is highly dynamic and populates multiple conformations, thus allowing binding of bisphosphorylated sequences to both SH2 domains. Regarding the mechanism of the allosteric transition leading to activation, our data indicated that phosphopeptide binding to the N-SH2 domain is possible both in the active and in the autoinhibited states, but it is significantly less probable in the latter. In addition, activation is coupled to the conformation of the N-SH2 loops, rather than to that of the central β-sheet. In this context, our simulations also indicate a previously unreported role of the BG loop in the allosteric mechanism.

## 2 Materials and methods

### 2.1 Replica-exchange molecular dynamics

The starting structures for the simulation of wild type in autoinhibited conformation (WT-INACTIVE) and mutant SHP2 in active conformation (E76K-ACTIVE) were taken from the RCSB Protein Data Bank (PDB code 4dgp and 6crf, respectively). Complete structures of WT-INACTIVE and E76K-ACTIVE were then obtained by modelling the missing residues and loops (WT-INACTIVE: 1–2, 236–245, 295–301, 314–323; E76K-ACTIVE: 90–92, 140–145, 154–165, 175–177, 203–209, 237–244, 313–324) in the original crystallographic structures with Modeller [59]. The starting structures for WT-ACTIVE and E76K-INACTIVE were instead built by amino acid substitution in the E76K-ACTIVE and WT-INACTIVE structure, respectively. The local side chain conformation of K76 (in E76K-INACTIVE) and E76 (in WT-ACTIVE) were optimized by selecting the most probable rotamer configuration [60].

GROMACS 2018.2 [61] was used to perform REMD simulations of each protein, solvated in a box of roughly 32,000 TIP4P-EW water molecules and with the appropriate counterions to neutralize net charges. Interactions between protein atoms were modelled by the AMBER99SB-ILDN force field [62]. The following equilibration protocol was used for each system: the potential energy was first minimized by using the steepest descent method; then, a 600 ps position-restrained with flexible water dynamics simulation were performed to rise the temperature from 10 K to 300 K. The resulting conformation was then used as starting point for all the replicas in REMD simulations. In REMD simulations, multiple replicas differing in temperature are run in parallel and the Metropolis criterion is used to exchange configurations between adjacent replicas at regular intervals. In this work, the system size demanded for 76 replicas to achieve a 10% exchange probability between replicas in the temperature range 300–400 K. The temperature scheme to cover the whole range was predicted on the basis of the system size [63]. Each replica was first equilibrated at the target temperature for 5 ns and finally, roughly 250 ns of REMD simulations were performed for WT-INACTIVE and E76K-INACTIVE and roughly 300 ns for WT-ACTIVE and E76K-ACTIVE. The frequencies of exchange between nearest-neighbour temperatures were in the interval 0.16–0.20 and the efficacy of the trajectories to sample all the different temperatures has been verified. The convergence of the production run was confirmed by calculating the root mean square deviation (RMSD) of all the possible pairs of structures in the 300 K ensembles [64], and the gyration radius of the whole protein and the secondary structure as a function of time (data not shown). Movies of the trajectories at 300 K are available as supplemental materials.

The trajectories were analysed with modules present in the GROMACS package. The core of the SH2 domains was defined according to [33], with slight changes to make it equivalent in the N- and C-SH2 domains (residues 6–8, 26–34, 41–47, 51–56, 63, 74–79, 97–101 for N-SH2 and residues 112–114, 132–140, 147–153, 167–172, 179, 190–195, 211–215 for C-SH2). For the PTP domain, residues with root mean square fluctuations (RMSF) lower than 0.15 nm in the WT-INACTIVE simulation were considered (residues 222, 223, 251, 255, 268, 282–292, 303–310, 326–334, 337, 338, 340–350, 352–356, 370–372, 380–383, 385–388, 390–393, 395–397, 400–404, 412–415, 417, 418, 420, 422–424, 428–443, 445, 452–468, 470–479, 486, 488, 490–494, 496–507, 509–511, 513–515, 517, 518, 520–522).

All analyses were carried out on the 300 K ensembles, by considering all the structures at that temperature, sampled every 200 ps. To assess the possible influence of the force field on the simulation results, we performed three independent plain dynamics simulations at 300 K, for two of our systems (WT-INACTIVE and E76K-ACTIVE) with the same conditions used in the REMD simulations, by using three different force fields: AMBER99SB-ILDN, CHARMM36m [65] and Amber ff19SB [66]. No significant differences between the three force fields were detected, regarding the mobility of the three protein domains observed in the simulations (data not shown). Structural figures were produced by using the UCSF Chimera package [67].

### 2.2 Reverse pulling of the Eck96 bis-phosphorylated peptide

The REMD trajectory of WT-ACTIVE was analyzed to search for configurations compatible with the binding of a bis-phosphorylated peptide. The binding of the high affinity peptide (Eck96 in the following) with sequence LN(pY)IDLDLV-Ahx_4_L-ST(pY)ASINFQK (where Ahx indicates 6-aminohexanoic acid) was tested [28]). The target configuration for WT-ACTIVE was selected among those that showed a binding-compatible distance (lower than 4 nm, see Fig. 4 and a reasonable relative orientation between the two SH2 domains. The possibility of binding was proven by a three-stage reverse pulling of Eck96 to the SH2 binding pockets: first the C-terminal segment (seg-1; sequence ST(pY)ASINFQK) and then the N-terminal part of the peptide (seg-2; sequence LN(pY)IDLDLV) of Eck96 were reverse pulled towards the C-SH2 and N-SH2 domain binding pockets, respectively; finally, the linker region was added to the system and pulled towards seq-1 and seq-2 termini. More in detail, in the first two stages, each peptide segment was initially set roughly 4 nm away from the center of mass of the corresponding SH2 domain, to prevent any interaction. The overall system was placed in a 12 × 18 × 15 nm box, was solvated with approximately 104,000 TIP3P water molecules and ions were added opportunely to neutralize the system. Position restraints were imposed on all the C_α_ atoms of SHP2-WT to ensure that the starting conformation was retained. The pull dynamics was performed using two distinct collective variables for each segment: the center-of-mass distances pY(seg-1)-R138(C-SH2) and +4 N(seg-1)-Y197(C-SH2); the center-of-mass distances pY(seg-2)-R32(N-SH2) and +5L(seg-2)-Y81(N-SH2). The reverse pulling forces (elastic constant force k = 1000 kJ mol^−1^ nm^−2^) guided the peptide segment into the SH2 domain binding pocket with a 0.001 nm/ps pace. The final structure with the two peptide segments bound to SHP2 was then used as starting point for the placement of the linker. A peptide bond between the latter and the C-terminus of the bound seg-2 was formed using the appropriate tools in UCSF Chimera 1.13 software package [67]. Subsequently, the C-terminus of the L residue of the linker was pulled towards the N-terminus of the S residue of seg-1 bound to the C-SH2 domain (using the same pulling parameters as the previous stages). Positions of C_α_ atoms in SHP2-WT were restrained and an additional harmonic potential (k = 1000 kJ mol^−1^ nm^−2^) was imposed to seg-1 and seg-2 atoms, to allow relaxation in their binding pocket during reverse pulling of the linker. Finally, a peptide bond between the linker and the N-terminus of seg-1 was formed and the obtained structure of the Eck96:SHP2-WT complex was energetically minimized. The stability of the complex was tested with 10 ns of simulations without restraints; in this case, the same conditions adopted in the REMD simulations were used.

## 3 Results

### 3.1 The active state of SHP2 is highly dynamic

To investigate the stability of the crystallographic conformation reported for the active state of the E76K variant, which can be considered as representative of the active state of SHP2 in the absence of stimuli, we performed REMD simulations of wild type SHP2 and of the E76K variant, starting from the X-ray structure 6crf (WT-ACTIVE and E76K-ACTIVE simulations). For comparison, the autoinhibited state was simulated, too (WT-INACTIVE and E76K-INACTIVE trajectories).

In all trajectories, the simulation protocol did not promote unfolding of individual domains, whose secondary structure was stably retained.

**Fig. 3:**
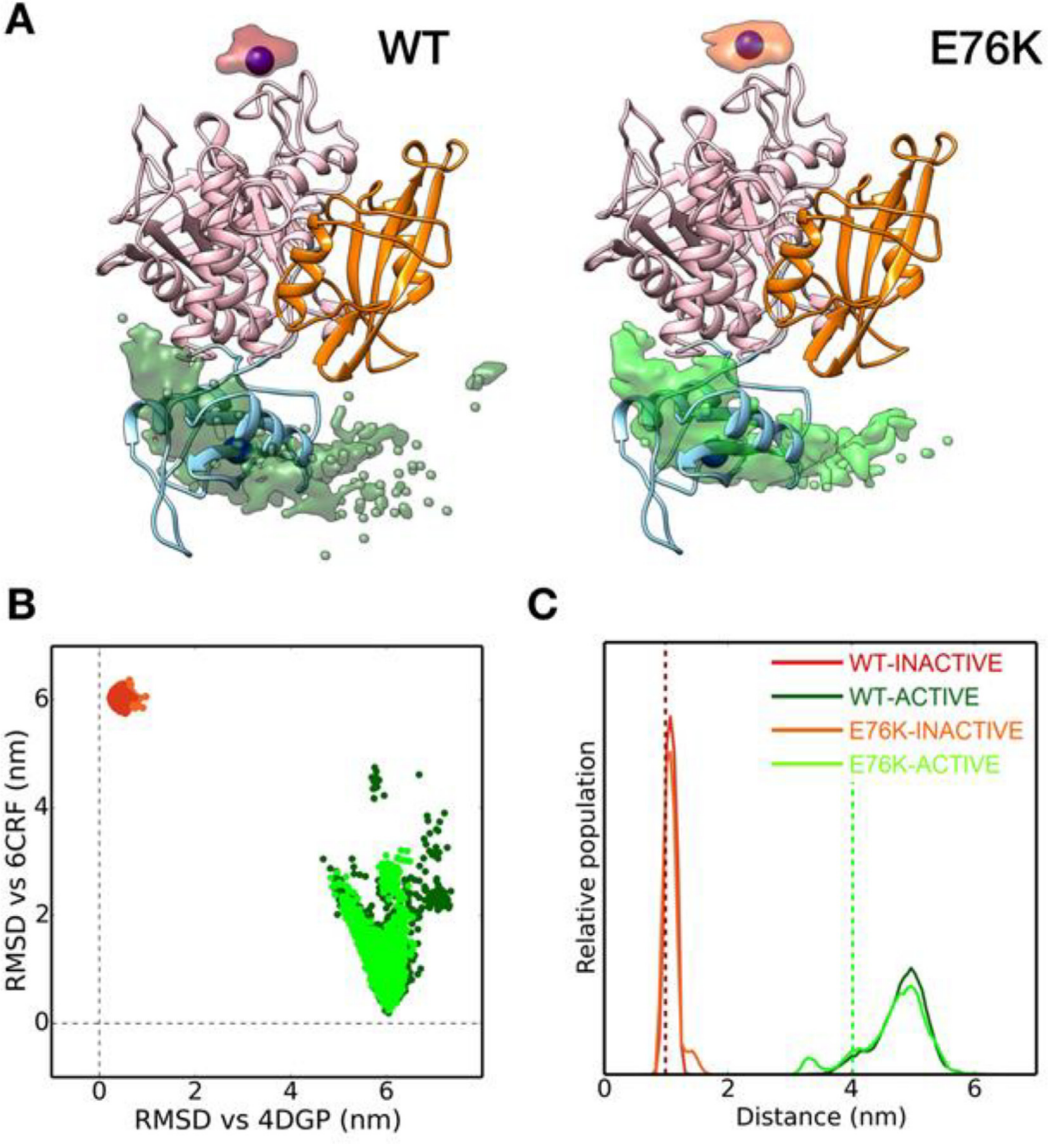
Conformational heterogeneity of SHP2. *Panel A)* Positions of the N-SH2 center of mass populated in the REMD simulations at 300 K of the autoinhibited (red) and active (green) states for both wild type SHP2 (darker colors) and the E76K variant (brighter colors) (see panel C for the color code); obviously, the N-SH2 domain is located in two opposite regions, with respect to the PTP domain, in the two states. As a reference, the 6crf structure is reported as a ribbon with N-SH2, C-SH2 and PTP domains colored in cyan, orange and pink, respectively. The N-SH2 center of mass positions in crystallographic structures of the inactive (PDB code 4dgp) and active (PDB code 6crf) states are shown as purple and blue spheres, respectively. The positions are calculated after removal of the roto-translational motions of the core of the PTP domain. *Panel B)* Backbone RMSD from the reference crystallographic structures after removal of the roto-translational motions of the core of the PTP domain. The same coloring scheme as in panel C) was used. *Panel C)* Distribution of distances between the center of mass of the N-SH2 blocking-loop (residues 58–62) and of the PTP catalytic loop (residues 454–467). Reference values from crystallographic structures are shown as vertical dashed lines, colored in red (inactive state, PDB code 4dgp) or green (active state, PDB code 6crf). (For interpretation of the references to color in this figure legend, the reader is referred to the web version of this article.)

Fig. 3A reports the position of the center of mass of the N-SH2 domain with respect to the PTP in the replicas at 300 K of the four simulations with respect to the PTP domain. In the simulations started from the active state, the N-SH2 domain did not retain its crystallographic configuration and sampled a wide ensemble of positions (green cloud in the figure). By contrast, the position of the N-SH2 domain remained essentially unchanged during the trajectories of the autoinhibited state.

No transitions between the active and inactive states were observed during the simulations. Fig. 3B shows the root mean square deviation (RMSD) of the N-SH2 backbone with respect to its position in the crystallographic active or inactive states, after removal of the roto-translational motions of the PTP domain. In agreement with the results reported in Fig. 3A, the simulations starting from the active state sampled a much wider conformational space with respect to that populated in the simulations of the autoinhibited state, with only minor differences between the wild type and E76K simulations. Furthermore, transitions between the conformational spaces sampled in the simulations started from the two states were absent, showing that the enhanced sampling allowed by the REMD protocol was unable to sample the activation transition, in line with the high energetic barrier determined experimentally [45], [46], [47], as discussed in the introduction.

Of note, the limited inter-domain dynamics in the inactive state did not affect the binding pocket of the proposed allosteric inhibitors [21], [50], which is located at the interface between PTP and N-SH2. Table S2 reports the distance between the Cα atoms of three residues lining the binding site of the allosteric inhibitors in the crystallographic structure of the complex between SHP2 and SHP099 (PDB code 5ehr). The distances remained essentially unchanged during the WT-INACTIVE and E76K-INACTIVE simulations.

Overall, the wild type and E76K proteins had a very similar behavior; a slight difference was observed between the simulations of the two proteins in the active state, where wild type SHP2 populated a few conformations where the detachment of the N-SH2 and PTP domain was more pronounced (this is illustrated in Fig. 3A by the presence of a small number of spots in a region completely separated from the rest of the green cloud).

To estimate the accessibility of the PTP active site, which modulates the catalytic activity, we followed the distance between the blocking loop (loop DE) on the N-SH2 domain and the catalytic loop in the PTP active site at 300 K (Fig. 3C). In the WT-INACTIVE simulation the distance remained very close to the crystallographic value. Even in the E76K-INACTIVE simulation a bona fide detachment of the blocking loop from the PTP domain was never observed. However, in this case, slightly larger distances (with values close to 1.5 nm) were populated, in agreement with the destabilization of the autoinhibited state induced by the E76K substitution [29], [32]. Very recently, based on targeted dynamics and single molecule fluorescence data, Tao et al. [46] proposed the existence of an intermediate in the activation transition pathway, close to the autoinhibited state and with a very limited activity, compared to the fully activated state [46]. Our data show that E76K-INACTIVE, but not WT-INACTIVE, populated conformers in which the PTP active site was slightly more accessible, possibly recapitulating the main features of the proposed intermediate. Fig. 3C describes the sampling of the same distance in the two simulations of the active state. Once again, the data show that in this case the protein is definitively more flexible than in the autoinhibited state, for both the wild-type protein and the E76K variant.

Finally, Fig. S2 reports the RMSFs of each residue during the four simulations, after removal of the roto-translational motions on the PTP domain. The much higher relative mobility of the N-SH2 domain (residues 3–104) in the active state, with respect to the autoinhibited state is clear also from this analysis. On the other hand, the relative mobility of the C-SH2 domain (residues 112–216) is not significantly affected by the activation transition. The same behavior, although obviously with a lower overall mobility, was observed in plain dynamics at 300 K performed with three different FFs (AMBER99SB-ILDN, CHARMM36m, and Amber ff19SB), suggesting that parametrization had only a marginal effect on our findings, if any.

### 3.2 The position of the N-SH2 domain in the active state is not stabilized by strong interdomain interactions

To explain the observed differences in domain mobility, we analyzed the interdomain ion-pair interactions in the four simulations. The results are summarized in Fig. S3. In the WT-INACTIVE simulation, the N-SH2/PTP interface was stabilized by different permanent interactions, including salt bridges involving residues R4/E252, D61/R465 and E76/R265, which were present in more than 60% of the conformations constituting the ensemble at 300 K (black lines in the figure) and E69/ K280 which was slightly less stable. These interactions, already present in the crystal structure (PDB code 4dgp), are spatially distant from each other, reflecting the wide region involved in the N-SH2/PTP interface in the autoinhibited state. In the E76K-INACTIVE simulation the stable interaction between residues E76 and R265 was, obviously, lost, but a less stable interaction between the residues R265 and E83 was formed, which was absent in the wild-type protein. Furthermore, the remaining salt-bridges observed in WT-INACTIVE were not significantly influenced by the E76K amino acid substitution. Even if a destabilization of the N-SH2/PTP interface in the E76K variant can be predicted, the persistence of these interdomain interactions in the E76K-INACTIVE simulation explains the experimental and simulative data available for this variant. While a distinct preference for the active state was demonstrated [29], [32], slow closed-to-open interconversion times were experimentally observed [45], [46], [47]. Differently from the large number of stabilizing interactions observed between the N-SH2 and PTP domains, only one interaction was detected between the two SH2 domains in the WT-INACTIVE and E76K-INACTIVE simulations, involving residues R5 and D192, in both cases. No ion-pair interactions were present between the C-SH2 and PTP domains.

The pattern of the interactions was completely different in the simulations of the active state. In this case, no interaction was stable in more than the 60% of the sampled conformations. In the crystallographic structure of the active state (PDB code 6crf) the N-SH2/PTP interface is significantly smaller than in the autoinhibited state. During both the WT-ACTIVE and E76K-ACTIVE simulations, despite the high mobility observed for the N-SH2 domain, this interface was almost never completely lost, with the only exception of a small number of conformations sampled in the WT-ACTIVE trajectory, as already discussed (Fig. 3A). The substantial stability of the interaction between the N-SH2 and PTP domains was witnessed by the partial persistence of two salt-bridges involving residues R4/D485 and R5/E225. At the same time, other stable interactions did not form. The apparent paradox of high inter-domain mobility and persistent interactions can be explained by visually inspecting the conformations constituting the ensemble at 300 K. R4 and R5 belong to an unstructured N-SH2 region. Thanks to its flexibility, this sequence behaves like a “hinge” between the two domains. N-SH2 rotates around this quite flexible hinge, by exploring different configurations with respect to the PTP domain, but it almost never loses these interactions completely.

Regarding the interactions involving the C-SH2 domain, the salt bridge between D192 and the N-SH2 residue R4 was lost in the simulations of the active state, but other interactions formed. Of note, no detectable ion-pair interactions between C-SH2 and PTP were observed in the active state simulations, either. Finally, only few interactions involve residues outsides the three domains, encompassing residues 110, 111 and 120.

### 3.3 Binding of bisphosphorylated peptides is possible in the conformations sampled in the active state

**Fig. 4:**
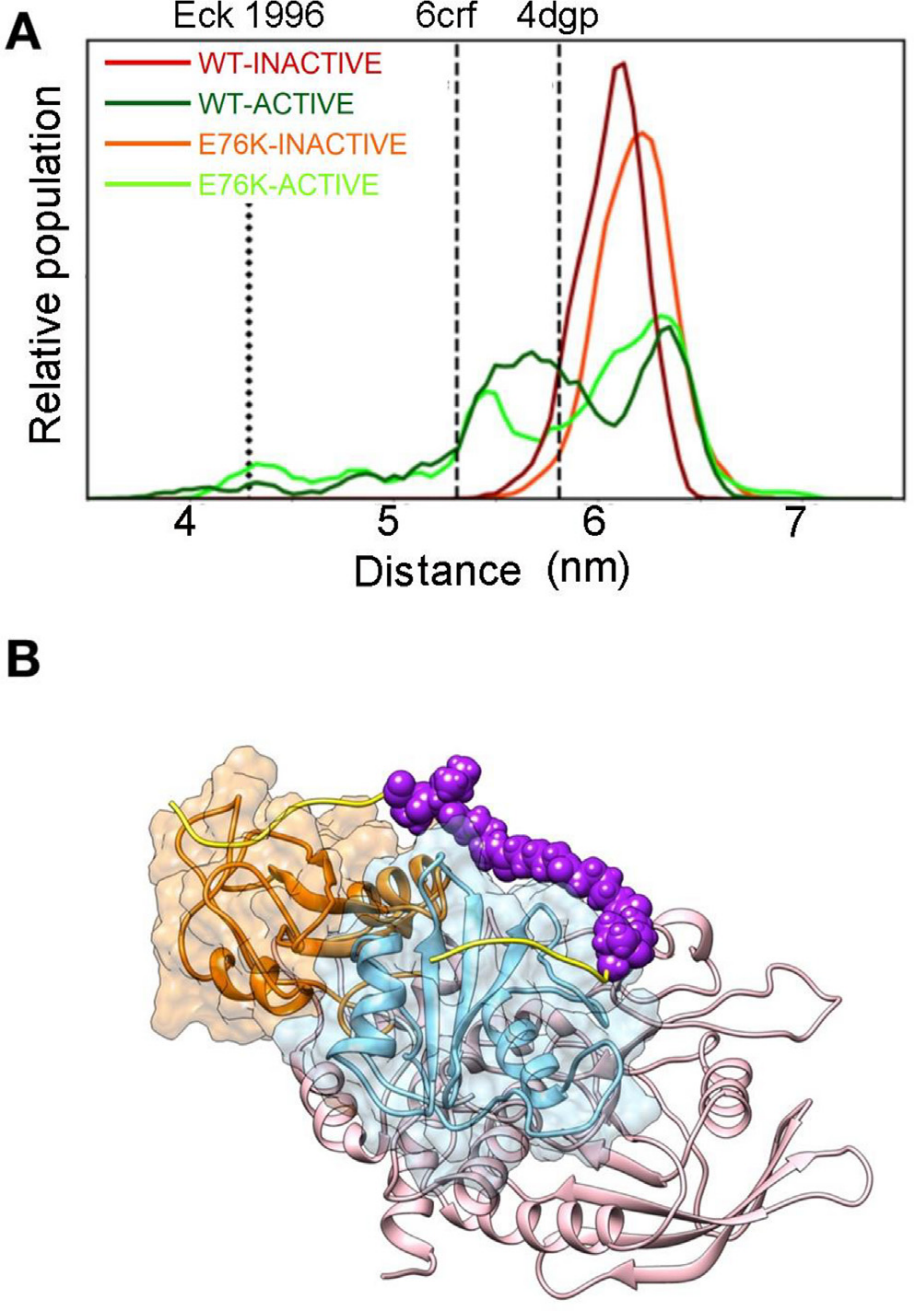
Interdomain arrangement and binding to bisphosphorylated sequences. *Panel A)* Distances between the C- and N- termini of the peptide fragments of Eck’s high affinity bisphosphorylated peptide bound to N-SH2 and C-SH2 domains. The plot reports distances between the C atom of the carboxyl group in the residue at position +5 (with respect to the pY) in the fragment bound to N-SH2 and the backbone amide atom of the residue at position −2 in the one bound to C-SH2. Values from crystallographic structures and for Eck’s peptide in the extended configuration are shown as vertical dashed lines. The same coloring scheme as in Fig. 3 was used. *Panel B)* Optimal configuration of SHP2/biphosphorylated peptide complex obtained by docking of the Eck’s peptide by inverse pulling method.

As discussed in the introduction, the crystallographic structure of the active state does not allow binding of both SH2 domains to a bisphosphorylated peptide of the optimal length, determined by Eck et al. [28]. However, our simulations showed significant variations in the interdomain arrangement, particularly in the position of the N-SH2 domain. Therefore, we analyzed our trajectories to understand whether the observed conformational flexibility leads to relative domain positions that would make association to bisphosphorylated sequences possible. For each conformation of the trajectories, we docked a phosphopeptide in each of the two SH2 domains. Successively, we measured the distance between the peptide termini that should be joined by the linker (Fig. 4A). Binding of a bisphosphorylated peptide of the optimal length is impossible in all the conformations sampled by the simulations of the autoinhibited state. By contrast, both simulations of the active state populate (although with a low probability) distances sufficient to connect the two phosphopeptide sequences with a linker. An example is provided in Fig. 4B. The stability of this complex was tested by performing a 10 ns long MD simulations without restraints. The peptide remained tightly bound to the protein, as witnesses by the stability of the interaction between the pY and the R residue at position β5 in the phosphate binding pocket (R32 and R138 in the N- and C-SH2 domains, respectively) [22]. During the simulation, the distance between the pY phosphorus and the arginine CZ atoms populated values of 0.43 ± 0.02 nm (N-SH2 domain) and 0.48 ± 0.02 nm (C-SH2 domain).

### 3.4 In the N-SH2 domain, but not in C-SH2, accessibility to the binding site increases in the active state

**Fig. 5:**
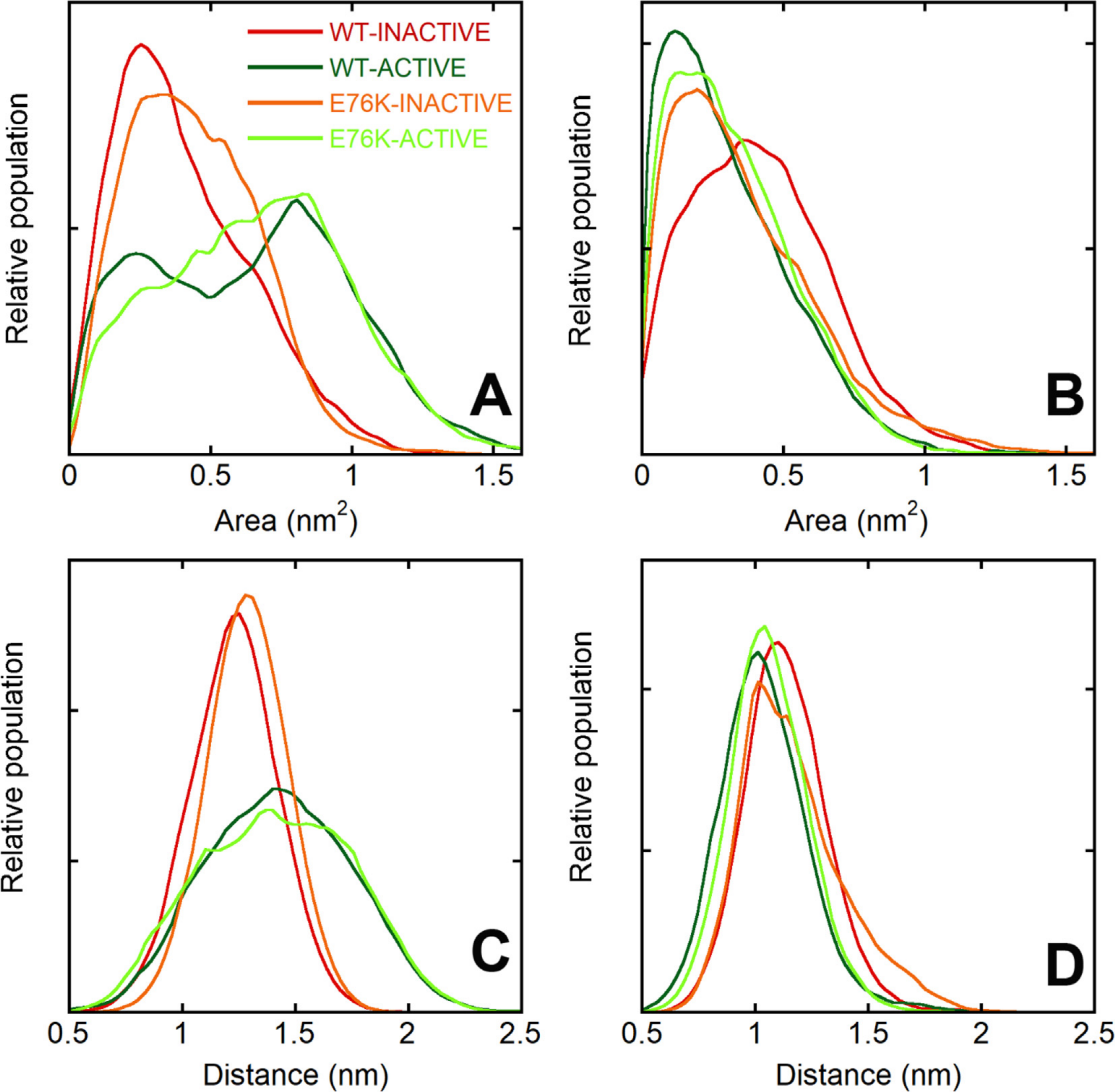
Accessibility of the N-SH2 and C-SH2 binding sites in the autoinhibited and active states. Top (A and B): SASA of the residues in the SH2 binding sites which interact with the C-terminal region (with respect to the pY residue) of the binding peptides (residues 54, 65 and 81 for N-SH2 and 170, 182 and 190 for C-SH2). Bottom (C and D): distances between the centers of mass of the backbone of the EF (residues 66–69 for N-SH2 and 182–185 for C-SH2) and BG (residues 84–96 for N-SH2 and 200–210 for C-SH2) loops, which regulate the accessibility to the binding site. Data from N-SH2 (left -A and C) and C-SH2 (right – B and D) are presented with the color code reported in panel A.

To clarify the allosteric mechanism regulating SHP2 function through its association with binding partners, we analyzed the possible coupling between activation and the accessibility of the SH2 binding sites. Fig. 5A and B report the solvent accessible surface area (SASA) of the pocket where phosphorylated sequences insert. A different behavior of the two SH2 domains was evident also in this case. In the N-SH2 domain, the binding site was significantly more accessible in the active than in the autoinhibited state. At the same time, the range of SASA values sampled in the simulations corresponding to the autoinhibited state was quite large and a considerable number of conformations reached values greater than 0.48 nm^2^, which correspond to the SASA of the considered residues in the X-ray structure of isolated domains (e.g. PDB 1aya). For comparison, the value in 4dgp (autoinhibited state) is 0.19 nm^2^. By contrast, for the C-SH2 domain, the SASA distributions were not affected by the activation transition.

These findings are consistent with experimental data showing that the N-SH2 domain binding affinity is highly enhanced by activation [29], [32], [34], [41]. In addition, they indicate that binding to the N-SH2 domain is possible in the autoinhibited state, too. The lower population of conformations showing a SASA greater than 0.48 nm^2^, in the autoinhibited simulations with respect to the active ones, is coherent with the significantly lower affinity of this state for the phosphopeptides.

### 3.5 Accessibility to the N-SH2 binding site is regulated by the EF and BG loops, whose motions are allosterically controlled by SHP2 activation

The access to the SH2 domain binding pocket is regulated by the conformation of the EF and BG loops. Fig. 5C and D report the distance between these two loops in the four simulations. For N-SH2, significantly larger values of this distance were sampled in the simulations WT-ACTIVE and E76K-ACTIVE, with respect to the corresponding simulations of the autoinhibited state. By contrast, no significant differences were observed for the C-SH2 domain. This behavior nicely parallels the SASA results, confirming that the dynamics of these loops controls the accessibility of the SH2 binding pocket.

**Fig. 6:**
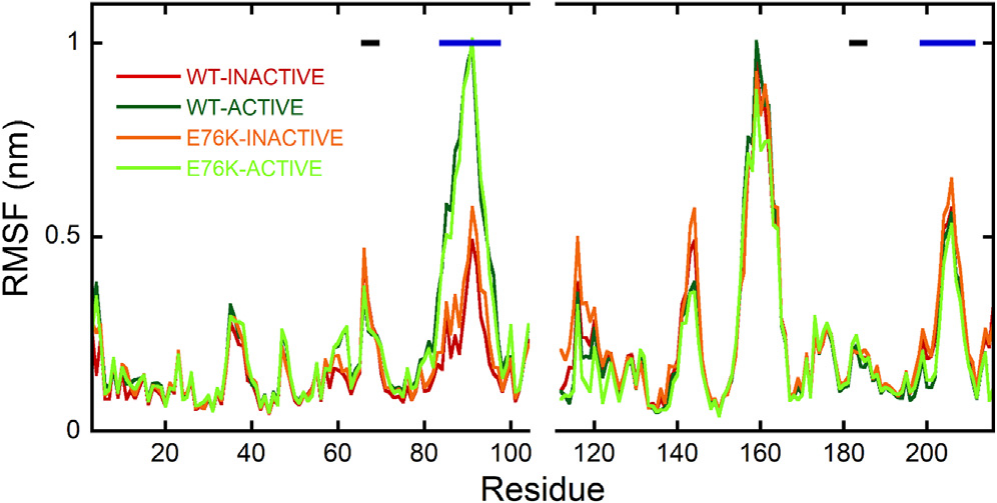
Mobility of SH2 loops. RMSF with respect to the average atom positions for N- and C- SH2 domains in the WT-INACTIVE, WT-ACTIVE, E76K-INACTIVE and E76K-ACTIVE trajectories at 300 K. Roto-translations have been removed on the C_α_ atoms of the residues in the core of each SH2 domain, considered individually. Black and blue horizontal lines represent residues belonging to EF and BG loops, respectively. The color code, reported in the figure, is the same as in Fig. 3. (For interpretation of the references to color in this figure legend, the reader is referred to the web version of this article.)

Fig. 6 reports the RMSF of the SH2 domain residues, after removal of the roto-translational motions of each domain considered individually, to focus on the internal dynamics. The BG loop of the N-SH2 domain was significantly more mobile in the WT-ACTIVE and E76K-ACTIVE simulations, with respect to WT-INACTIVE and E76K-INACTIVE trajectories. By contrast, fluctuations of the EF loop were quite similar in the four simulations. These data suggest a previously unrecognized role of the BG loop dynamics in the SHP2 allosteric regulation mechanism. Overall, our simulations clearly demonstrate a correlation between the conformation and dynamics of the EF and BG loops and the active/autoinhibited states of SHP2, contrary to what was proposed recently [39], based on simulations starting from the autoinhibited state, only.

### 3.6 Y66 is a key residue controlling the open/closed transition of the N-SH2 binding site

**Fig. 7:**
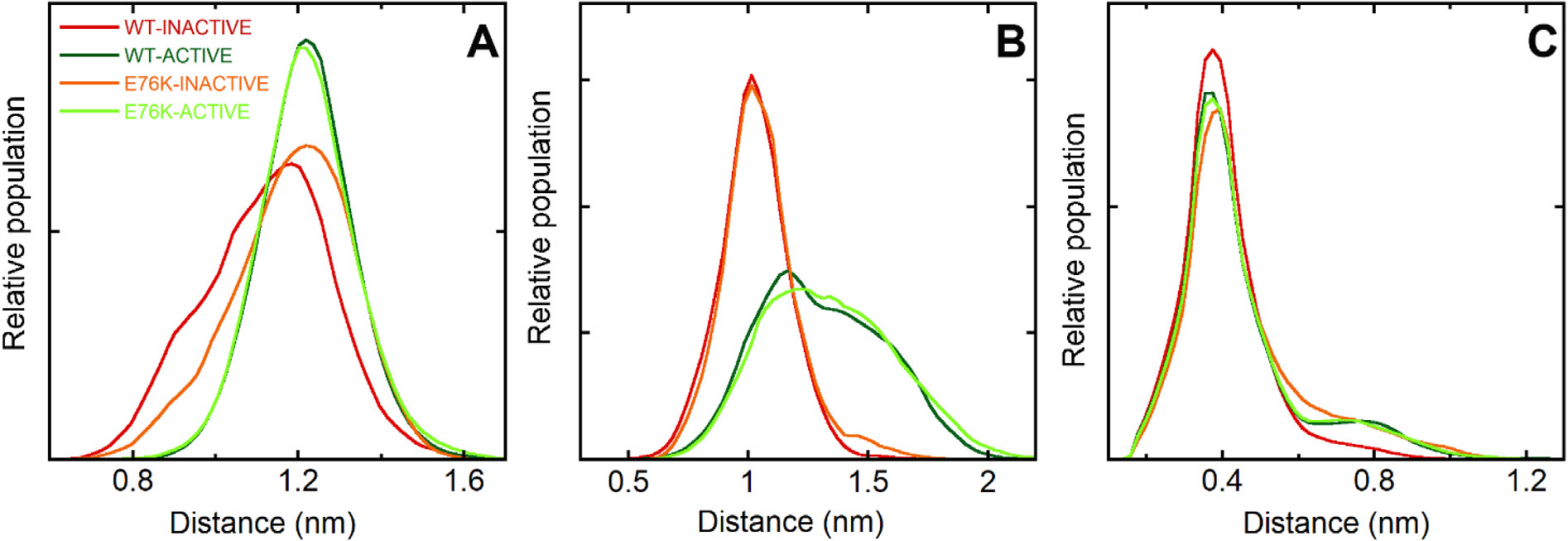
Dynamics of the N-SH2 EF and BG loops and of the central β-sheet. Normalized distributions of the distances between: A) the C_α_ atoms of I54 and of the EF loop (residues 66–69); B) the C_α_ atoms of I54 and of the BG loop (residues 84–96). C) The carbonyl C atom of G39 and the backbone N atom of N58. This distance represents the opening of the central β-sheet, according to Anselmi and Hub [39]. Data refer to the WT-INACTIVE, WT-ACTIVE, E76K-INACTIVE and E76K-ACTIVE simulations at 300 K and the color code, reported in panel A, is the same as in Fig. 3.

**Fig. 8:**
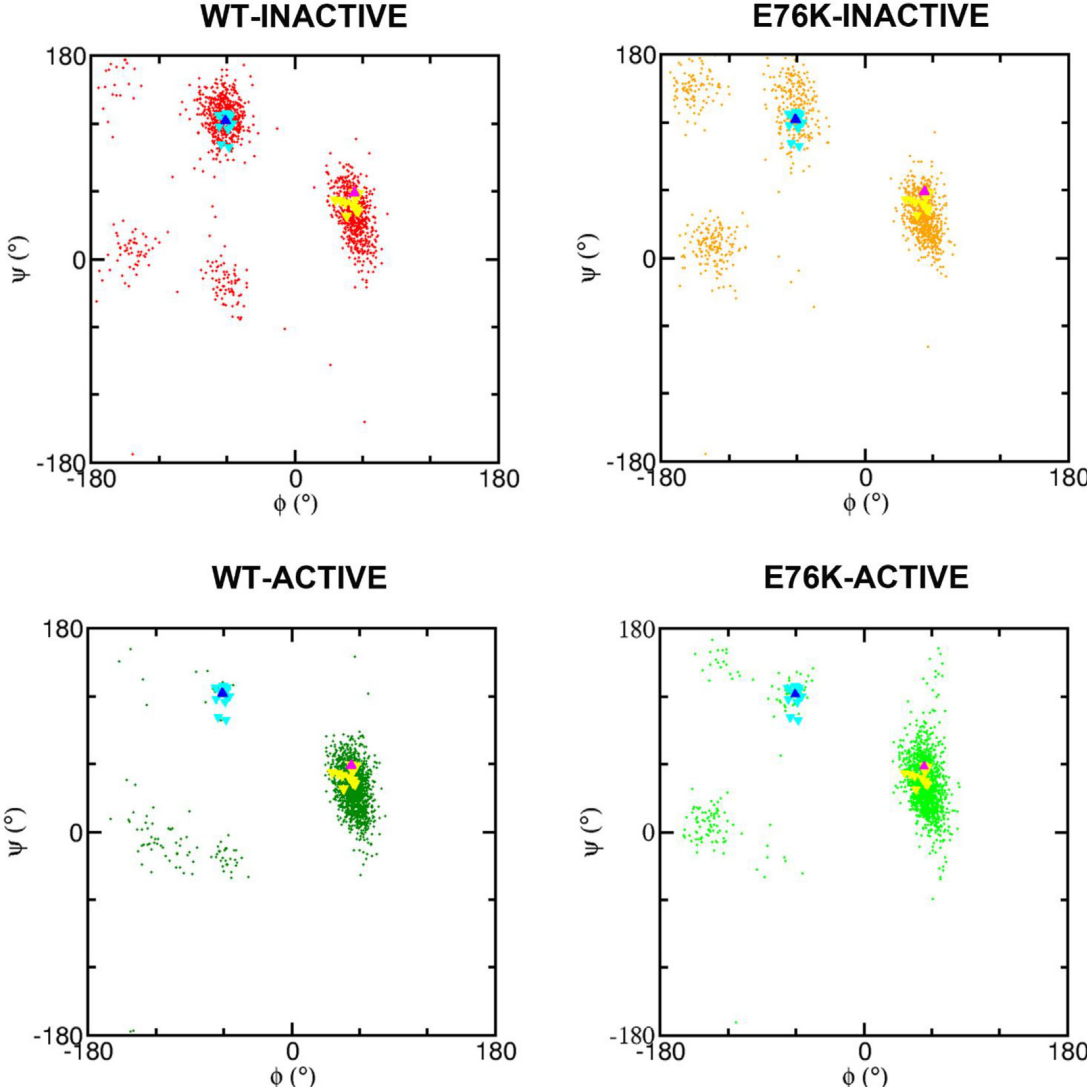
Local backbone conformation of Y66 in the autoinhibited and active states. Ramachandran plots at 300 K of Y66 from REMD simulations. In all panels, values derived from crystallographic structures have been reported, for comparison. Structures of free N-SH2 domains are shown as yellow triangles (PDB 1aya, 1ayb, 1ayc, 1ayd, 3tkz, 3tl0, 4je4, 4qsy, 5df6, 5x7b, 5x94, and the N-SH2 structure from [28]), E76K SHP2 in the active state is shown as a magenta triangle (PDB 6crf) structures for complete SHP2 in the inactive state are reported in cyan (PDB 4dgp, 4dgx, 4ohd, 4dhe, 4ohh, 4ohi, 4ohl, 5ehp, 5ehr, 6cmp, 6cms, 7emn) and blue triangles (PDB 2shp). (For interpretation of the references to color in this figure legend, the reader is referred to the web version of this article.)

Opening of the N-SH2 domain binding site can be caused by motions of the EF loop, the BG loop, or both. To investigate the contribution of the EF loop, we followed the distance of the center of mass of its backbone with respect to residue I54, which is part of the binding site and belongs to the core of the domain (Fig. 7A). A bimodal distribution was evident in both the WT-INACTIVE and E76K-INACTIVE simulations, while only larger values were populated in the corresponding simulations of the active state. These data indicate that the EF loop is always open in the active state, while it opens only transiently in the autoinhibited state. As discussed in the introduction, MacKerell and coworkers hypothesized that the dynamics of EF loop is mainly determined by the conformation of Y66 [30]. The many crystallographic structures available today, including that of the whole protein in the active state, are still consistent with this hypothesis. As shown in Fig. 8, in the crystallographic structures of the autoinhibited state, the backbone of Y66 is always in the region corresponding to extended conformations, while in isolated N-SH2 domains, or in the structure of the active state, the ϕ and ψ angles of Y66 fall in the left-handed helical region. In our simulations, too, Y66 populates predominantly the helical region of the Ramachandran plot in the simulations of the active state. The situation is more complex and dynamic in the trajectories of the autoinhibited state, with Y66 populating both extended and helical-like conformations. These findings are consistent with the data on the accessibility of the binding pocket (Fig. 5), confirming that the conformation of the EF loop is regulated by the activation transition, but that the binding site of the N-SH2 domain is transiently open even in the autoinhibited state. This latter finding makes an induced fit mechanism possible for SHP2 activation, with phosphopeptides able to bind to the N-SH2 domain in the autoinhibited state of SHP2, rather than to the active conformation only (as in the conformational selection mechanism). In addition, our findings support a critical role of Y66 in the conformational transition of the N-SH2 domain.

### 3.7 The BG loop behaves differently in the inactive and active state

Regarding the BG loop of the N-SH2 domain, Fig. 6 highlighted its higher mobility in the active state. To investigate the effect of this different flexibility on the accessibility of the N-SH2 binding site, similarly to the EF loop, we calculated the distances between the C_α_ atoms of the BG loop and that of I54 in the four simulations (Fig. 7B). In both simulations of the active state, larger distances were populated, with respect to those of the autoinhibited state, indicating that the different dynamics of the BG loop contributes to the higher accessibility of the N-SH2 binding site in the active state. This contribution of the BG loop was not previously considered, and at first it might be surprising, considering that this region is not directly in contact with the other domains in any of the available crystallographic structures. However, the BG-loop connects the B-helix (residues 74–83) and the G strand (residues 100–102), which are in contact with the PTP and C-SH2 domains, respectively. As a consequence, the different dynamics of the BG loop could be caused by the different relative mobility of the three domains in the autoinhibited and active states. For instance, Fig. S3 shows that salt-bridges involving residues of the B-helix are present in the simulations of both WT and E76K in the inactive state (between R265 on PTP and E76 or E83 on N-SH2) but they are lost in the active state simulations. To quantify the effect of the changes in the network of salt-bridges on the relative mobility of the PTP and N-SH2 domains in this region, Fig. S4 reports the minimum distance between E83 and the PTP domain. E83, which is located at the N-terminus of the B-helix, immediately before the BG-loop, remains in contact with the PTP domain in a wide portion of the conformations sampled in the two simulations of the autoinhibited state, and it is generally closer to PTP than in the simulation of the active state. Slight differences can be observed between the E76K-INACTIVE and WT-INACTIVE simulations, probably due to the different stability of the already mentioned salt bridge between E83 and R265, in the two cases.

### 3.8 The conformation of the central β-sheet of the N-SH2 domain is not correlated to the active/autoinhibited state

As discussed in the introduction, based on simulations starting from the autoinhibited structure, [39] proposed that the conformation of the central β-sheet of the N-SH2 domain, rather than of the EF and BG loops, correlates with the activation status of SHP2 (i.e., active or autoinhibited state). To test their hypothesis, taking advantage of our characterization of the active state, we report in Fig. 7C the distance between the carbonyl C atom of G39 and the backbone N atom of N58, used by Anselmi and Hub, to measure the opening of the β-sheet, during the simulations for the WT protein and the E76K variant in the autoinhibited and active states. The dynamics of the central β-sheet was essentially identical in the four simulations, and it was not influenced by the active/autoinhibited state of the protein.

## 4 Discussion

The conformational transition controlling the activation of SHP2 has been the object of multiple studies over the years. Several simulative approaches have been used to reach the open, catalytically active conformations of the protein. Before the crystallographic structure of the active state became available, MacKerell and coworkers simulated SHP2 activation by evaluating the potential of mean force (PMF) along the distance between the centers of mass of the N-SH2 and PTP domains [31], starting from the autoinhibited crystallographic structure. By pulling the two domains far from each other, this approach creates conformations in which the two domains are totally dissociated, in contrast with the compact interdomain arrangement in the crystallographic structure of the active state [37]. In a recent study, Anselmi and Hub [39] used a similar approach and evaluated the PMF along the distance between the centers of mass of the backbone atoms of the N-SH2 blocking loop and of the catalytic PTP loop, again starting from the inactive crystallographic structure These simulations sampled only the first step of SHP2 activation, i.e., the release of the N-SH2 domain from the PTP active site. The conformations obtained in the simulation were quite different from the crystallographic structure of the active state, with the N-SH2 maintaining an orientation very similar to the one it has in the autoinhibited conformation. Tao and colleagues [46], took into account the crystallographic structure of the active state for the first time, and followed SHP2 activation by using that structure as a target conformation. This approach is based on the assumption that the available X-ray structures fully represent the conformations populated in the autoinhibited and active states in solution, and therefore it is not suitable to assess the reliability of these structures.

As discussed in the introduction, several lines of evidence indicated that the crystallographic structure of the active state does not fully represent the conformational ensemble of activated SHP2. In the present study, we exploited the enhanced sampling ensured by the REMD approach to analyze the conformations actually populated in solution in both states, without any bias other than the available X-ray structures, used as starting points. Our results confirmed that the compact structure observed in the crystal for the active state is not conserved in solution and a wide ensemble of conformations is accessible, in which the N-SH2 domain assumes different positions with respect to the PTP catalytic domain, with which it always remains in contact. SHP2 single amino acid substitutions stabilizing the putative inter-domain interface in the active state have never been observed in patients, while the majority of pathogenic, activating amino acidic substitutions destabilizes the autoinhibited state [32]. These two kinds of mutations should have similar effects on the equilibrium between active/autoinhibited states, and therefore on protein function. A highly dynamic active state explains this evidence. The multiple interdomain arrangements populated in solution also make binding of bisphosphorylated sequences to the two SH2 domains possible, while this was not feasible in the crystallographic structure. Finally, the ensemble of structures deriving from our simulations is in better agreement with solution SAXS data than the crystallographic structure 6crf (Fig. S5).

In our simulations we did not observe any transition between the autoinhibited and active states. However, this behavior was expected, considering the experimentally observed interconversion times (in the seconds time frame [45], [46], [47]) and the maximum temperature used in our REMD protocol (400 K), selected to avoid significant unfolding of the protein. Our goal was to characterize the two states separately, rather than to sample the activation transition. As already discussed, attempts in this direction had to assume an active state where the two domains are completely dissociated, or corresponding to the crystallographic structure. Our simulations indicate that both these assumptions are unjustified. Simulating SHP2 activation remains challenging.

We observed a very similar behavior for WT SHP2 and its E76K variant. However, E76K is biochemically and functionally markedly different from WT SHP2 [29]. The pathogenicity of the E76K substitution is due to a marked shift of the activation equilibrium towards the active state, which for this mutant is predominantly populated even in the absence of stimuli. As discussed above, our simulations cannot characterize this effect, but they suggest that the mutation shifts the equilibrium without modifying the main features of the two states. In the active state, residue 76 is not involved in inter-domain interactions (see Fig. S3) and the substitution only marginally affects the inter-domain arrangement or dynamics. On the other hand, in the autoinhibited state, E76 is involved in an interdomain salt-bridge, which is lost in the mutant. Therefore, a destabilization of this conformation was expected. Even if we did not observe a complete detachment of the N-SH2 blocking loop from the PTP catalytic site in the E76K-INACTIVE simulation (due to the high energetic barrier, as discussed above), distances higher than 1.5 nm were populated (Fig. 3C), indicating a loosening of the inter-domain interaction in the mutant. Interestingly, the existence of an intermediate state in the SHP2 activation pathway, with features very similar to the autoinhibited state, has been recently proposed by Tao and co-workers [46] based on simulative and experimental results.

The conformational ensembles of SHP2 in the inactive and active states allowed us to analyze the key conformational features involved in the allosteric activation mechanism. Our data demonstrated a clear correlation between the active/inactive state of SHP2 and the accessibility of the N-SH2 domain binding site, which was significantly larger in the active state. By contrast, the accessibility of the binding site of the C-SH2 domain was not influenced by the activation status of the whole protein. These findings are qualitatively consistent with previously available crystallographic data, where the N-SH2 binding groove is closed in structures of autoinhibited SHP2, and open in the X-ray structure of the active state or in all those of the isolated N-SH2 domain. However, the simulations also showed that the N-SH2 binding site partially opens in the autoinhibited state, too (Fig. 3), even if to a lower extent and with a lower frequency than in the active state. This effect is mainly due to the dynamics of the EF loop, which in the autoinhibited state populates both closed and open conformations in the simulations of the autoinhibited state, while the BG loop remains always closed in this state. The fact that autoinhibited structures with an open N-SH2 binding site have never been observed in the crystal state is possibly caused by significant constraints induced by crystallographic contacts on the loops that govern the accessibility of the N-SH2 binding site [39].

Our findings on SH2 binding site dynamics are relevant for the different models proposed for the mechanism of SHP2 activation by association to bisphosphorylated sequences through its SH2 domains. Experimental data have clearly demonstrated that association of a phosphopeptide to the N-SH2 domain only is sufficient for activation of the phosphatase [20]. Based on these findings, the C-SH2 domain is considered to contribute to the overall binding affinity and selectivity for specific bisphosphorylated peptides, without a direct involvement in the activation mechanism. Our observation that no correlation exists between the SHP2 activation status and the conformation of the C-SH2 domain binding site supports this idea.

By contrast to the C-SH2 domain, the N-SH2 domain plays a critical role in SHP2 activation. Binding of phosphorylated peptides to this domain causes a population shift towards the active state. At the same time, mutations that favor the active state by destabilizing the autoinhibited conformation also induce a higher affinity of the N-SH2 domain for its binding partners [41]. MacKerell and coworkers [30], first proposed a possible allosteric mechanism connecting the N-SH2/PTP interaction with the opening/closing of the N-SH2 phosphopeptide binding cleft. They suggested a critical role of the Y66 residue, which links the conformation of the DE loop (interacting with the PTP active site) and of the EF loop (controlling the access to the N-SH2 binding groove). More in detail, a left-handed helical-like backbone conformation for Y66 would correspond to an open cleft, while and an extended-like backbone conformation would result in a closed N-SH2 phosphopeptide binding site. Based on PMF calculations starting from the autoinhibited state of SHP2, recently Anselmi and Hub proposed an alternative allosteric mechanism [39], where SHP2 activation correlates with the conformation of the central β-sheet in the N-SH2 domain, rather than with the conformation of the loops controlling access to the binding site. Our data clearly discriminate between these two hypotheses. We did observe a clear correlation of the activation status with the accessibility of the N-SH2 binding groove, controlled by loops EF and BG, and coupled to the Y66 conformation, in agreement with the MacKerell model. By contrast, the activation status had no effect on the conformation of the central β-sheet. The discrepancy of these findings with the results reported by the Hub group can be rationalized by considering that their simulations did not sample the active state, but only a possible intermediate, induced by the variable used in their PMF calculations. In addition, it is worth mentioning that their model does not explain the experimentally observed higher affinity of the N-SH2 domain for its binding partners in the active state.

Interestingly, our simulations identified a previously undescribed role of the BG loop in the allosteric process. This aminoacidic stretch is predominantly unresolved in the X-ray structures, where it is located far from the PTP and C-SH2 domains. Therefore, this loop had not been considered relevant in the models discussed above. In our simulations, we observed that the BG loop is much more flexible in the active state, and overall, this higher mobility favors access to the binding site. We attribute this behavior to the large interdomain motions in the active state, which affect the B-helix and the G strand and, in turn, the BG loop. This effect could not have been revealed by comparing X-ray structures or simulations of autoinhibited SHP2 and of the isolated N-SH2 domain.

Finally, our data provided indications on how phosphopeptide binding induces the activation transition. As discussed in the introduction, two alternative mechanisms have been proposed also in this case, which can be associated to the classical induced fit and conformational selection models. In the first case, a phosphopeptide binds to the N-SH2 domain in the autoinhibited state and induces a conformational transition that causes its dissociation from the PTP active site. In the second hypothesis, an equilibrium between the autoinhibited and active states exists already under basal conditions, although it is shifted towards the inactive conformation. However, in the active state the N-SH2 domain has a higher affinity for phosphorylated sequences, and therefore phosphopeptide binding to N-SH2 stabilizes catalytically active SHP2. As already discussed, our data showed a higher N-SH2 binding site accessibility in the active state, in agreement with the conformational selection model. However, we observed that the binding cleft opens more rarely also in the autoinhibited state, so that the induced fit mechanism cannot be ruled out and the hypothesis of a multistep binding mechanism (with pY associating first and favoring accessibility of the binding pocket to the rest of the phosphopeptide sequences) is probably unnecessary. The occurrence of conformations with a partially open N-SH2 binding site in the inactive state is in contrast with the available crystallographic structures, where the binding site is always closed in the autoinhibited state. However, other recent simulations of the inactive state reported similar results and showed that a closed N-SH2 binding site in the X-ray structures could be induced by crystallographic contacts [39]. Thermodynamically, the two mechanisms are equivalent (the phosphopeptide-bound/active-state is favored with respect to the phosphopeptide-bound/autoinhibited-state, and the unbound/autoinhibited-state is favored with respect to the unbound/active-state), and they could both concur to SHP2 activation.

## 5 Conclusion

In this study, we presented REMD simulations of SHP2 in the active and in the autoinhibited state. Using crystallographic structures as the starting point for simulating the two states, we could avoid the bias induced by steered simulations used in previous studies, and thus we were able to characterize various aspects of the SHP2 regulation mechanism:

• the crystallographic structure of the active state is unstable in solution, where the N-SH2 domain is highly mobile, leading to interdomain arrangements that allow binding of bisphosphorylated sequences to the two SH2 domains;
• in the active state, we observed no differences between the wild-type protein and the E76K variant. In the autoinhibited state conformations with a nascent dissociation of the N- SH2/PTP domains were slightly populated in the E76K protein;
• the activation status (active or autoinhibited state) is strongly correlated with the accessibility of the N-SH2 binding site, which is higher in the active state, and is controlled by the EF loop (through a conformational switch triggered by Y66) and by the BG loop (whose different mobility in the active and inactive states indicates an unpredicted role of this region in SHP2 regulation). By contrast, and contrary to a recent proposal (Anselmi et al., 2021), the conformations populated by the central N-SH2 β-sheet are the same in the active and autoinhibited states of SHP2. This structural feature does not appear to play a key role in the activation mechanism;
• in crystallographic structures of the autoinhibited state, the binding site of the N-SH2 domain is always inaccessible. By contrast, in solution it can open transiently even in the inactive state (although with a lower probability than in the active state). As a consequence, both induced fit and conformational selection allosteric mechanisms are possible.

## CRediT authorship contribution statement

**Paolo Calligari:** Conceptualization, Methodology, Investigation, Visualization, Formal analysis, Writing – review & editing. **Valerio Santucci:** Investigation, Visualization, Formal analysis. **Lorenzo Stella:** Conceptualization, Methodology, Visualization, Formal analysis, Supervision, Funding acquisition, Project administration, Writing – original draft, Writing – review & editing. **Gianfranco Bocchinfuso:** Conceptualization, Methodology, Visualization, Formal analysis, Supervision, Funding acquisition, Project administration, Writing – original draft, Writing – review & editing.

## Declaration of Competing Interest

The authors declare that they have no known competing financial interests or personal relationships that could have appeared to influence the work reported in this paper.

## Appendix A Supplementary data

Supplementary video 2 E76K-ACTIVE

Supplementary video 3 E76K-INACTIVE

Supplementary video 4 WT-ACTIVE

Supplementary video 5 WT-INACTIVE
