# Supplementary material for: Discriminating between competing models for the allosteric regulation of oncogenic phosphatase SHP2 by characterizing its active state
Source: Comput Struct Biotechnol J. 2021 Nov 3;19:6125–39. doi: 10.1016/j.csbj.2021.10.041 (PMC8632847; doi:10.1016/j.csbj.2021.10.041)
Supplement: Supplementary data 1 [file mmc1.pdf]

SUPPLEMENTARY MATERIAL for

Allosteric regulation of a crucial oncogenic target: unbiased simulations of the autoinhibited and active states of SHP2 phosphatase

Paolo Calligari, Valerio Santucci, Lorenzo Stella, Gianfranco Bocchinfuso
Dipartimento di Scienze e Tecnologie Chimiche, Università di Roma Tor Vergata.

Table S1: Articles reporting MD simulations of SHP2 relevant for the present work.

Reference	Method	Simulated system and starting state	Brief synopsis (relevant for the present paper)
Wieligmann et al.. In Silico Biol 2002;2:305	Plain MD simulations	Isolated N-SH2 domain in ligand-bound and free states	Description of the allosteric mechanism leading to domain dissociation upon phosphopeptide binding. The dynamics of the N-SH2 loops is discussed
Tartaglia et al. Am J Hum Genet 2006;78:279	Plain MD simulations	Whole protein in the autoinhibited state	The closed state is perturbed by pathogenic mutations
Bocchinfuso et al. Proteins Struct Funct Bioinf 2007;66:963	Plain MD simulations	Whole protein in the autoinhibited state	The closed state is perturbed by pathogenic mutations
Guvench et al. BMC Struct Biol 2007;7:1	Plain and constrained dynamics to evaluate PMF profiles	Whole protein (in the autoinhibited state) and isolated N-SH2 domain	N-SH2 allosteric mechanism: role of the N-SH2 loops and of Y66.
Darian et al. Proteins Struct Funct Bioinf 2011;79:1573	Plain and constrained dynamics to evaluate PMF profiles	Whole protein in the autoinhibited state	PMF profile along the distance between the centers of mass of the N-SH2 and PTP domains for different pathogenic SHP2 variants
Martinelli et al. Hum Mol Genet 2008;17:2018	Plain MD simulations	Isolated N- and C-SH2 domains	Effect of single pathogenic amino acid substitutions on the dynamics of the SH2 domains
Martinelli et al. J Biol Chem 2012;287:27066	Plain MD simulations	Isolated N-SH2 domain	Effect of single pathogenic amino acid substitutions on the dynamics of the N-SH2 domain
Li et al. J Mol Liq 2016;223:509	Solvent perturbed MD simulations	Whole protein in the autoinhibited state	Study of SHP-2 allosterism

Pannone et al. Hum Mutat 2017;38:451–	Plain MD simulations	Whole protein in the autoinhibited state	Effect of single pathogenic amino acid substitutions on the regulatory mechanism of SHP2
Li et al. J Biomol Struct Dyn 2018;36:3856	Plain MD simulations	Whole protein in the autoinhibited state	Study of the allosterism of SHP2 and effect of single amino acid pathogenic substitutions
Rehman et al. J Chem Inf Model 2019;59:3229	Plain MD simulations	Whole protein in the autoinhibited state	Effect of single amino acid pathogenic substitutions on PTP activity
Wang et al. Comput Biol Chem 2019;78:133	Plain MD simulations	Whole protein in the autoinhibited state	Stability of the autoinhibited state in wild type SHP2 and single point pathogenic variants
Martinelli et al. Hum Mutat 2020;41:1171	Plain MD and REMD simulations	Whole proteins or truncated constructs lacking the N-SH2 or both the N- and C-SH2 domains	Effect of single pathogenic amino acid substitutions on the regulatory mechanism of SHP2
Wang et al. Front Chem 2020;8:1059	Plain MD simulations	Whole protein (in the autoinhibited state) and isolated SH2 domains, also in the presence of phosphopeptides	Association between SHP2 and its binding partners, and allosteric regulation mechanism of SHP2, focusing on the dynamics of N-SH2 loops
Anselmi et a. J Chem Inf Model 2020;60:3157	Plain MD simulations	Isolated N-SH2 domain, also complexed with phosphopeptides	Interactions stabilizing the N-SH2/phosphopeptide complex and effect of binders on the dynamics of the N-SH2 domain
Anselmi and Hub. Sci Rep 2020;10:1	Plain MD simulations, umbrella sampling dynamics, pulling dynamics, restrained dynamics	Whole protein in the autoinhibited state and isolated N-SH2 domain	Conformations of the N-SH2 domain in the presence of phosphopeptides and their effects on SHP2 activation. . Role of the central β-sheet

Wang et al. Molecules 2021;26:837	Plain MD simulations	Whole protein (in the autoinhibited state) and isolated SH2 domains, also in the presence of phosphopeptides	Study of the allosteric regulation mechanism of SHP2
Anselmi and Hub. Proc Natl Acad Sci 2021;118: e2025107118	Plain MD simulations, umbrella sampling dynamics, pulling dynamics, restrained dynamics	Whole protein in the autoinhibited state, both in solution and in the crystal. Isolated N-SH2 domain.	N-SH2 allosteric mechanism
Tao et al. J Biol Chem 2021;296: 100538	Targeted dynamics by adiabatic biased molecular dynamics (ABMD), between the autoinhibited and active states	Whole protein	Study of SHP2 activation; an intermediate state between autoinhibited and active states is proposed.
Marasco et al. Comput Struct Biotechnol J 2021;19:2398	Plain MD simulations	Isolated N- and C-SH2 domains, also complexed with phosphopeptides	Allosteric transitions in the N-SH2 domain. A preliminary interaction between the pY and the N-SH2 domain is proposed as the first binding event.

Table S2: Stability of the SHP099 binding pocket. Distances between the C_α atoms of residues R111, T219 and E249 in the X-ray structure of SHP2 in the presence of the SHP099 allosteric inhibitor (PDB code 5ehr) and in the 300 K ensemble of WT-INACTIVE and E76K-INACTIVE REMD simulations. The three residues line the binding pocket for SHP099.

Pair Residue	X-ray distance (nm)	distance in the WT-INACTIVE simulation (nm)	distance in the E76-INACTIVE simulation (nm)
R111 - T219	1.0	1.2 ± 0.3	1.2 ± 0.2
R111 - E249	1.0	0.8 ± 0.2	0.9 ± 0.2
T219 - E249	1.2	1.3 ± 0.2	1.3 ± 0.1

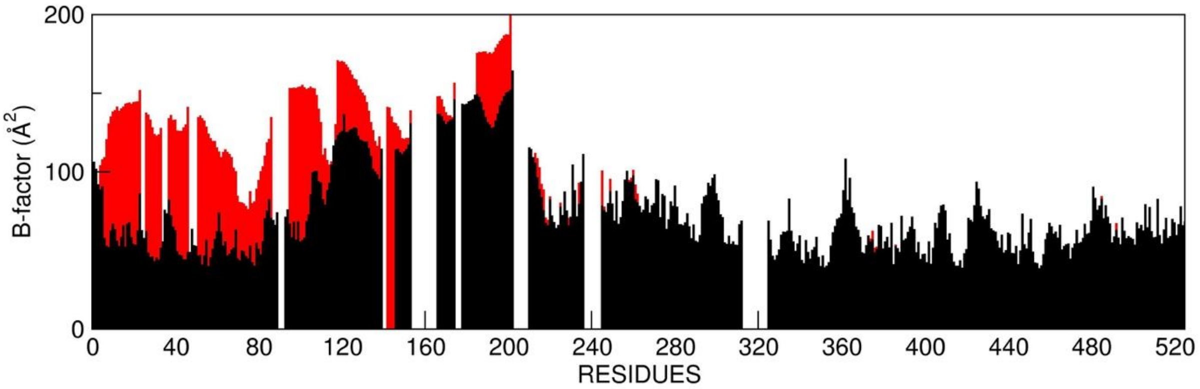

Fig. S1. Crystallographic Debye-Waller factor of SHP2 in the active conformation (PDB code 6crf). Debye-Waller factors (B-factor) of the two protein chains (chain A and B are shown in black and red, respectively) found in the same crystallographic asymmetric unit. Chain B clearly presents a higher B-factor for the whole N-SH2 domain and for some residues of C-SH2.

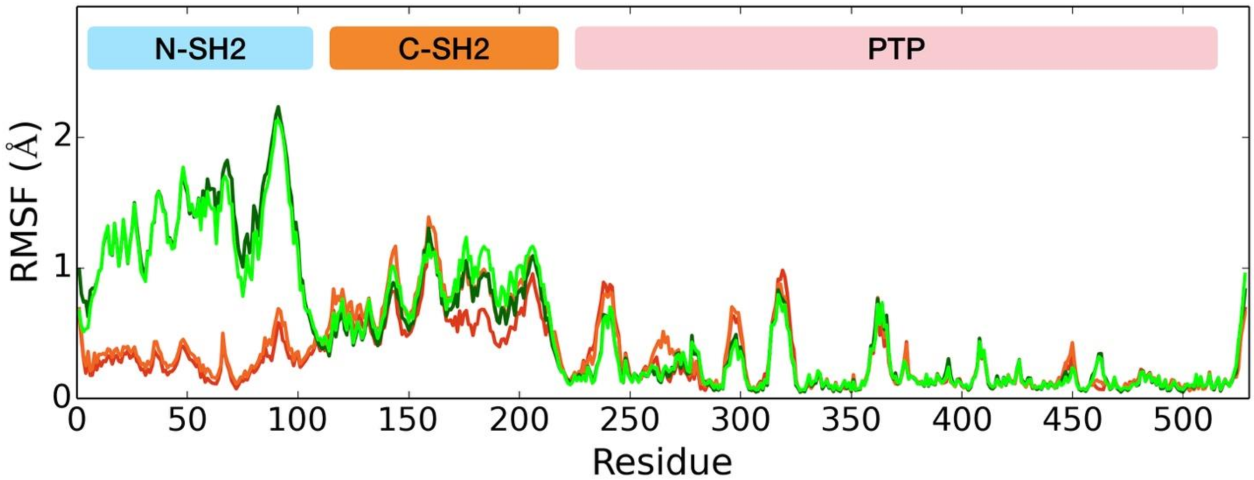

Fig. S2. Mobility of the SH2 domains with respect to the PTP domain. RMSF with respect to the average atom positions for the SHP2 residues in the WT-INACTIVE, WT-ACTIVE, E76K-INACTIVE and E76K-ACTIVE trajectories at 300 K, after removal of the roto-translations on the backbone of the PTP domain**.** The color code is the same as in Fig. 3 in the main text. Domain boundaries are indicated on top.

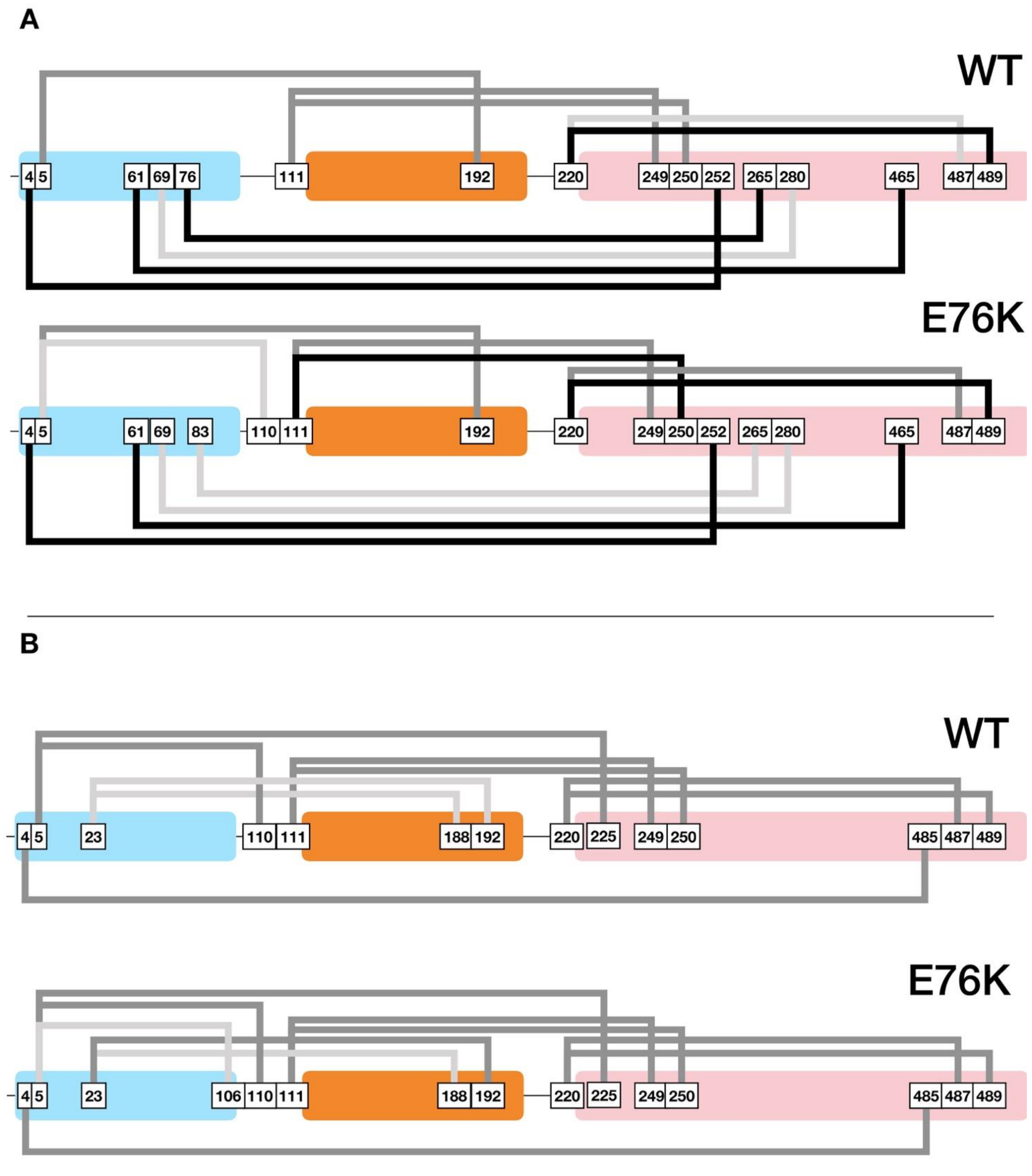

Fig. S3. Interdomain ion-pair interactions. Schematic representation of the inter-domain ion-pair interactions found in REMD simulations of the inactive (*panel A*) and active state (*panel B*) at 300 K. Linkers between residues are colored according to the persistence of the interaction (light grey: interaction presents in less than 30% of the conformations; dark grey: between 30% and 60%; black: greater than 60%).

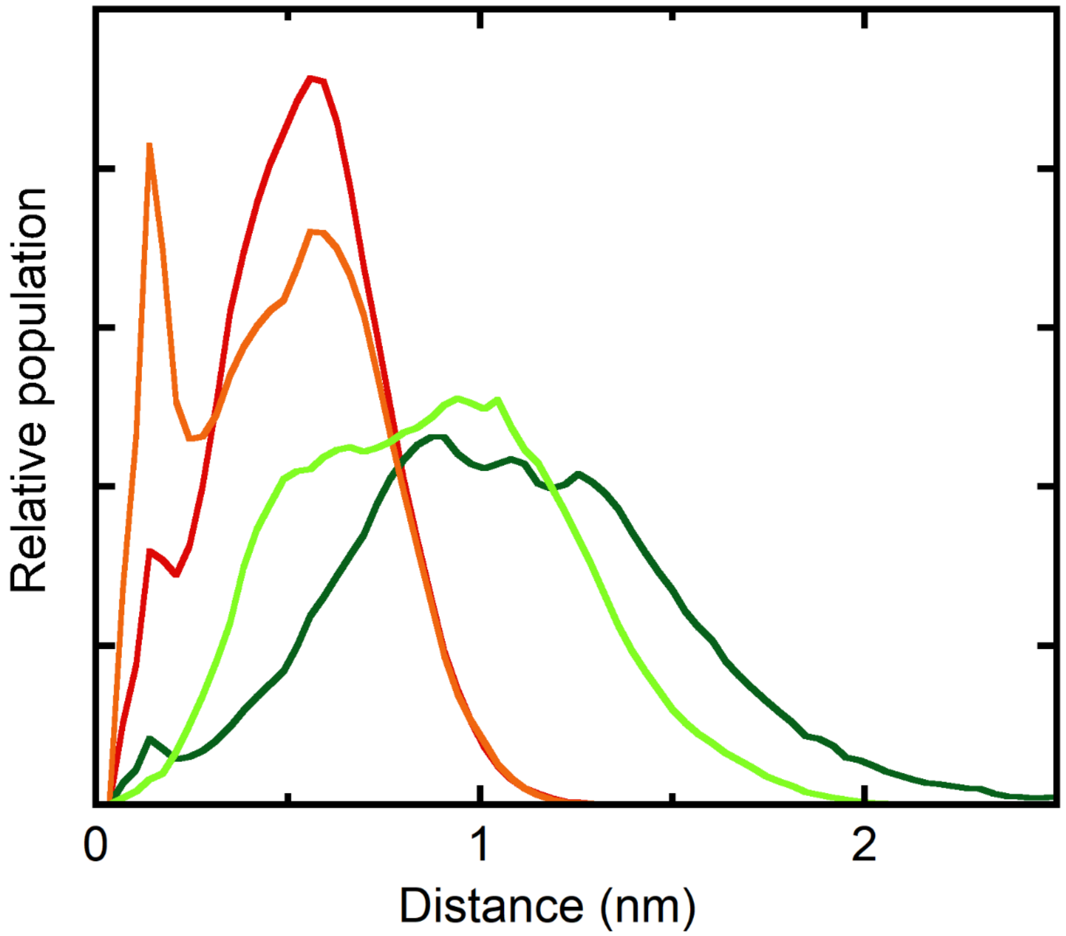

Fig. S4. Interaction between the PTP domain and the N-SH2 B-helix. Distribution of the minimum distances between E83 and the PTP domain (residues 221-524) during the WT-INACTIVE, WT-ACTIVE, E76K-INACTIVE and E76K-ACTIVE simulations at 300 K. The color code is the same as in Fig. 3 in the main text.

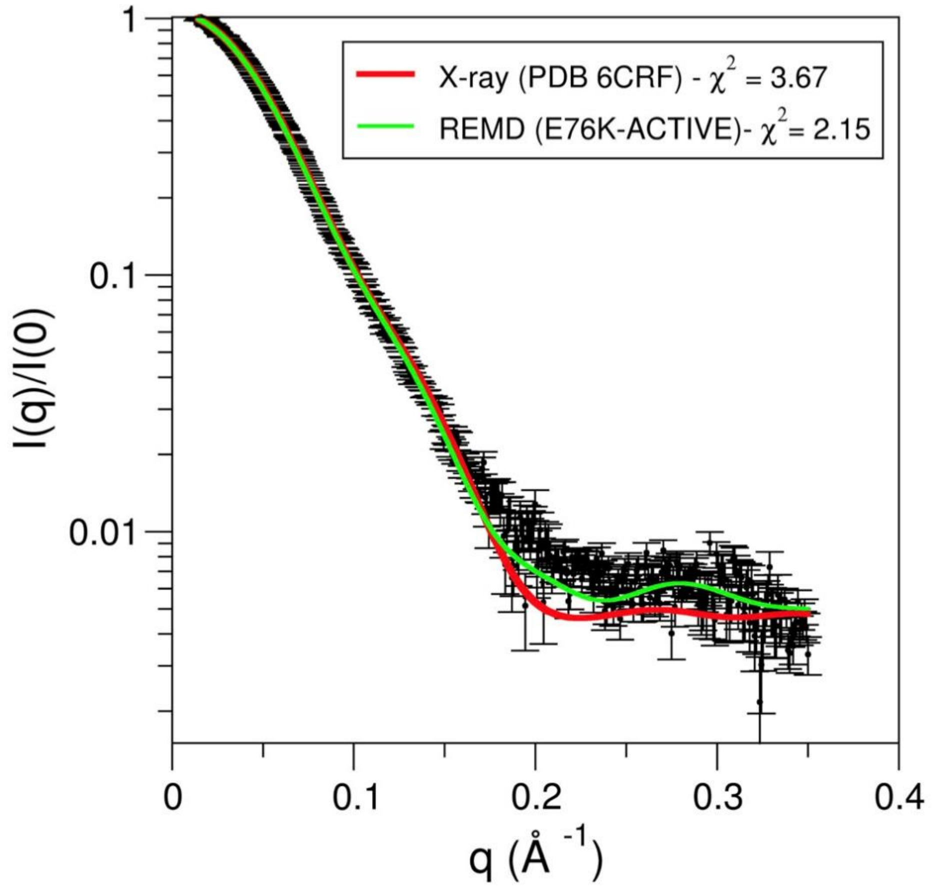

Fig. S5. Comparison between SAXS data and the REMD simulation for the active state. The SAXS profile calculated from the conformations explored in the E76K-ACTIVE trajectory (green line) is compared to experimental data from [Pádua et al. Nat Commun 2018;9:1], for the E76K protein in solution, by a multi-conformation fit performed in MultiFOXS [Schneidman-Duhovny et al. Biophys J 2013;105:962]. For comparison, the profile calculated for the 6crf crystallographic structure is reported, too (red line).
